# Tumor-associated macrophages in cancer: recent advancements in cancer nanoimmunotherapies

**DOI:** 10.1186/s13046-022-02272-x

**Published:** 2022-02-19

**Authors:** Nisha Kumari, Seung Hong Choi

**Affiliations:** 1 Department of Radiology, Seoul National University Hospital, Seoul National University College of Medicine, 101, Daehak-ro, Jongno-gu, Seoul, 03080 South Korea; 2 Center for Nanoparticle Research, Institute for Basic Science (IBS), Seoul, 00826 Republic of Korea; 3 School of Chemical and Biological Engineering, Seoul National University, Seoul, 00826 Republic of Korea

**Keywords:** Tumor-associated macrophages, Tumor microenvironment, Carcinogenesis, Macrophage repolarization, Nanomaterials, Nanoimmunotherapies

## Abstract

Cancer immunotherapy has emerged as a novel cancer treatment, although recent immunotherapy trials have produced suboptimal outcomes, with durable responses seen only in a small number of patients. The tumor microenvironment (TME) has been shown to be responsible for tumor immune escape and therapy failure. The vital component of the TME is tumor-associated macrophages (TAMs), which are usually associated with poor prognosis and drug resistance, including immunotherapies, and have emerged as promising targets for cancer immunotherapy. Recently, nanoparticles, because of their unique physicochemical characteristics, have emerged as crucial translational moieties in tackling tumor-promoting TAMs that amplify immune responses and sensitize tumors to immunotherapies in a safe and effective manner. In this review, we mainly described the current potential nanomaterial-based therapeutic strategies that target TAMs, including restricting TAMs survival, inhibiting TAMs recruitment to tumors and functionally repolarizing tumor-supportive TAMs to antitumor type. The current understanding of the origin and polarization of TAMs, their crucial role in cancer progression and prognostic significance was also discussed in this review. We also highlighted the recent evolution of chimeric antigen receptor (CAR)-macrophage cell therapy.

## Background

Cancer immunotherapy has emerged as a breakthrough approach in cancer treatment for eliminating minimal residual tumors by activating the inherent capacity of the immune system and improving the survival of advanced-stage patients [1, 2]. Although clinical trials have achieved promising outcomes, there are still certain issues to be addressed, such as low clinical rates, steady rates, immune-related side events, and unusual clinical reactions [3]. To achieve a long-lasting, efficacious antitumor response, cooperation between innate and adaptive immunity is advantageous. Immune cells in cancer patients are not only generally ineffective against cancer cells but also actually encourage tumor development, which reduces the therapeutic efficacy of standard treatments [4, 5]. Among all other innate cells, macrophages are a vital part of the innate immune system and are crucial in normal homeostasis, inflammation, and phagocytosis [6]. The high ratio of macrophages in cancers has been thought to be a mechanism involved in anticancer surveillance [7]. However, several studies have demonstrated that macrophages might act as “bad guys” in oncogenesis and neoplastic development by boosting genetic instability and angiogenesis while suppressing the immune response and cancer cell apoptosis [8].

Based on morphological, phenotypical and functional heterogeneity, macrophages are categorized into two distinct subtypes: M1 and M2 macrophages. M1 macrophages play a crucial role in antitumor immunity and primarily mediate proinflammatory processes in the tumor microenvironment (TME), whereas M2 macrophages have been demonstrated to have protumor features and to promote tumor growth and metastasis [9]. M2 macrophages, along with a small population of M1 macrophages, are called “tumor-associated macrophages” (TAMs), the most diverse immune cells in the TME and critical for tumor growth [9, 10]. Tumor cells secrete chemokines and growth factors to attract macrophages and transform them into the protumorigenic M2 type. The prognostic significance of TAM infiltration is associated with poor clinical outcomes in various cancers, which reduces the response to standard treatments [11, 12]. Furthermore, considerable dynamic changes in macrophage subpopulations were also found to be associated with the efficacy of immunotherapy [13–15]. Therefore, as a new-brand target, researchers are becoming increasingly interested in modulating TAMs for therapeutic purposes.

Nanotechnology is a multidisciplinary scientific research field that focus on various type of nanomaterials as well as on the use of innovative nano-devices in the numerous fields of interest [16]. Nanotechnology enables the detection of tumor at early stage which help to reduce the number of patients with advanced stages of malignancies [17]. With recent developments in nanotechnology, researchers can manipulate nanomaterials to bind to specific receptors, that are overexpressed in tumors, improving specificity and sensitivity which results in better tumor detection [18]. Various types of nanomaterial-based contrast agent such as super magnetic iron oxide (SPIO) and ultra-small super magnetic iron oxide (USPIO) have a longer blood circulation half-life and can recognize unique cell surface markers which results in better MRI contrast properties and have better clarity and accuracy, that helps accurate tumor diagnosis [19, 20]. Specifically engineered nanomaterials administers chemotherapy precisely to the tumor which prevents the drug from causing toxicity to the normal cells surrounding the tumor, enhances the efficacy of radiotherapies and leads to better curative effects [21]. Nanoparticles offers modifiable features such as size, shape, charge, surface and functional properties and this customization can be utilized synergistically with precision medicine therapies to improve patient’s stratification methods, indicates that nanoparticles are approaching the era of precision medicine [18, 22]. Nanoimmunotherapies are nanomaterial-based drug formulations that can improve the therapeutic effects of immunotherapies by focusing on immunosuppressive microenvironment and thus activates the immune system by interacting with other immune cells. With recent nanobiotechnological advancements, nanomaterials have received considerable interest in tumor immunotherapy because of their advantages in targeted delivery, precise locational drug release, simple surface functionalization, combination therapy and low immunogenicity with excellent performance in the activation of the immune system [23, 24]. In fact, targeted drug delivery systems based on a variety of nanomaterials have immensely transformed the fields of TAM-related immunotherapies [25, 26]. These nanomaterials can improve the therapeutic effect of immunotherapies by focusing on the immunosuppressive microenvironment and thus activate the immune system by interacting with other immune cells accompanied by reduced off-target toxicity and immune-related adverse events. Several studies have shown that nanoparticles induce the repolarization of anti-inflammatory M2-type macrophages towards a proinflammatory M1 phenotype, which is associated with tumor inhibition in various cancers [27, 28]. However, TAMs has also been reported to serve as drug depot, that accumulates significant nanoparticles which allows the local delivery of nanotherapeutics to nearby tumor cells and increase their efficacy by changing the spatial diffusion of drugs within tumors. Researchers showed that the uptake of nanoparticles by tumor macrophages was an important mechanism for accumulation of the drug to be delivered into the tumor in a therapeutically beneficial manner and depletion of macrophages resulted in significant decreases of tumor nanoparticle deposition and makes the treatment less effective [29]. By utilizing the synergistic benefits of TAMs and nanomaterials, TAM-targeting nanoimmunotherapies are realizable, feasible, and several macrophage-targeting nanomedicines have been established in recent years.

In our review, we discussed the current understanding of TAM origin, their heterogeneity and plasticity in TME and how macrophage activation and polarization can be controlled and altered for targeted therapeutic purposes, followed by the crucial role of macrophages in cancer progression. We also highlight the recent emergence of chimeric antigen receptor (CAR)-macrophage therapy. The main focus of our review is to describe the current nanomaterial-based potential therapeutic strategies that target TAMs in tumors, including restricting the survival of M2-type TAMs, inhibiting their recruitment toward tumors and functional repolarization of tumor-supportive M2-type TAMs to tumoricidal M1-type TAMs. This article is expected to aid the understanding of recent research progress in material-mediated modulation of the macrophage immune response and advance macrophage-related applications in cancer nanoimmunotherapy.

## Main text

### Origin and polarization of TAMs

The exact origin of TAMs has always been a topic of controversy. However, with modern lineage tracing technologies, our understanding of TAM origin has changed considerably; thus, TAMs may have at least four origins: a) F4/80^high^ macrophages originate from yolk sac, b) F4/80^low^ macrophages develop from bone marrow [5, 30], c) Langerhans cells are derived from fetal liver [31] and d) some proportion of TAMs originate from extramedullary hematopoiesis [32]. The large proportions of tissue-resident macrophages (TRMs) are initially originated from embryonic progenitors such as yolk sac and fetal liver that seed tissues during the prenatal and perinatal periods and gives rise to locally proliferating, self-maintained TRMs that can persist into adulthood [5, 33]. The mononuclear phagocytic system develops from the primitive ectoderm of yolk sac and gives rise to macrophages without monocytic precursors. Following this primitive system, definitive hematopoiesis occurs in fetal liver, which is initially seeded by hematopoietic progenitors from yolk-sac. The hematopoiesis in the fetal liver diminishes when bone marrow hematopoiesis begins, and hematopoiesis stem cells (HSCs) in the bone marrow and spleen become the primary source of circulating monocytes [34]. In bone marrow, the macrophage lineage differentiates into granulocytes-monocyte precursors, monocytes and dendritic cell precursors, pre-monocytes, monocytes and then into macrophages precursors [35]. In some organs, the embryonic macrophages are swiftly replaced by HSCs-derived monocytes and in some such as brain, macrophage subsets like microglia appears to have an embryonic origin with limited contribution from HSCs under homeostatic conditions [36, 37]. The pancreas, breast and lung are among the tissues that contain macrophages of mixed origin [38, 39]. Researchers revealed that embryonic and monocytic macrophages may have different phenotypes and functions in tumors cells [39, 40]. TAMs are generally assumed to derived from circulating monocytes, although 50% of the macrophages in brain, lungs and pancreatic cancer in mice were found to come from tissue-resident populations [40, 41]. Although, the interplay between TAMs of various origins has yet to be fully understood, but investigations in mice models can give some evidence. Recent reports demonstrated the increased accumulation of tissue-resident macrophages within tumor cells during the initiation of tumor formation, and their depletion led to reduced tumor growth in lung carcinoma [42]. Recently, Etzerodt et al. showed that a distinct subgroup of CD163^+^ Tim4^+^ resident omental macrophages was responsible for metastasis in ovarian cancer and that their depletion reduced tumor progression [43]. While both BMDMs and TRMs are seen in tumors, various tumors are likely to contain varying quantities of both. It is also unclear whether TRMs within tumors maintain tissue-specific transcriptional determinants. Further research is required to determine whether TRMs and BMDMs have similar effects on tumor growth, and alternative treatment methods are needed to regulate or deplete these two populations. However, it is evident that decreasing the macrophage population is required to diminish the production of immunosuppressive cytokines in tumors. Moreover, the diversity of TAMs origins contributes to the complexity of the TME; therefore, understanding the origin of TAMs is essential for selecting TAM-targeting strategies. Furthermore, the expression of chemoattractants in the TME determines the quantity and kinds of monocytes recruited from the circulation. As already mentioned, TAMs are a heterogeneous population that includes resident cells of embryonic origin present at birth and invading monocytes/macrophages recruited during early carcinogenesis by chemokines secreted by tumor and stromal cells (such as CCL2, CCL5, and CSF-1) [44]. Initially, based on the various stimulating factors and secreted products, macrophages can be traditionally divided into two categories: classically activated M1 macrophages stimulated by lipopolysaccharides, interferons, granulocyte macrophage colony stimulating factor (GM-CSF), and tumor necrosis factor (TNF-α), which facilitate a proinflammatory response against disease; and alternatively activated M2 macrophages stimulated by IL-4 and IL-13, which eventually activate the JAK/STAT pathway to induce the production of anti-inflammatory cytokines, further expediting tumor progression by rebuilding the TME [45]. However, M1/M2 categorization is too simple due to its vast diversity. In fact, TAMs resemble M2 macrophages and are identified as the M2 type [46], although they do not have any specific markers. In contrast, the polarizing cell surface markers for M1 macrophages (CD86, CD16, CD64, TLR2, CD120b, and SLAMF7) and M2 macrophages (CD23, CD1a, CD1b, CD163, CD226, and CD93) are distinct [47]. Numerous studies have shown the involvement of TAMs in tumor development, highlighting the need to better understand the mechanism of TAMs chemotherapeutic agent interactions to predict the efficacy of standard therapies and to develop therapeutic strategies that enhance the antitumor response of TAMs [48].

### TAMs in cancer development

**Fig. 1:**
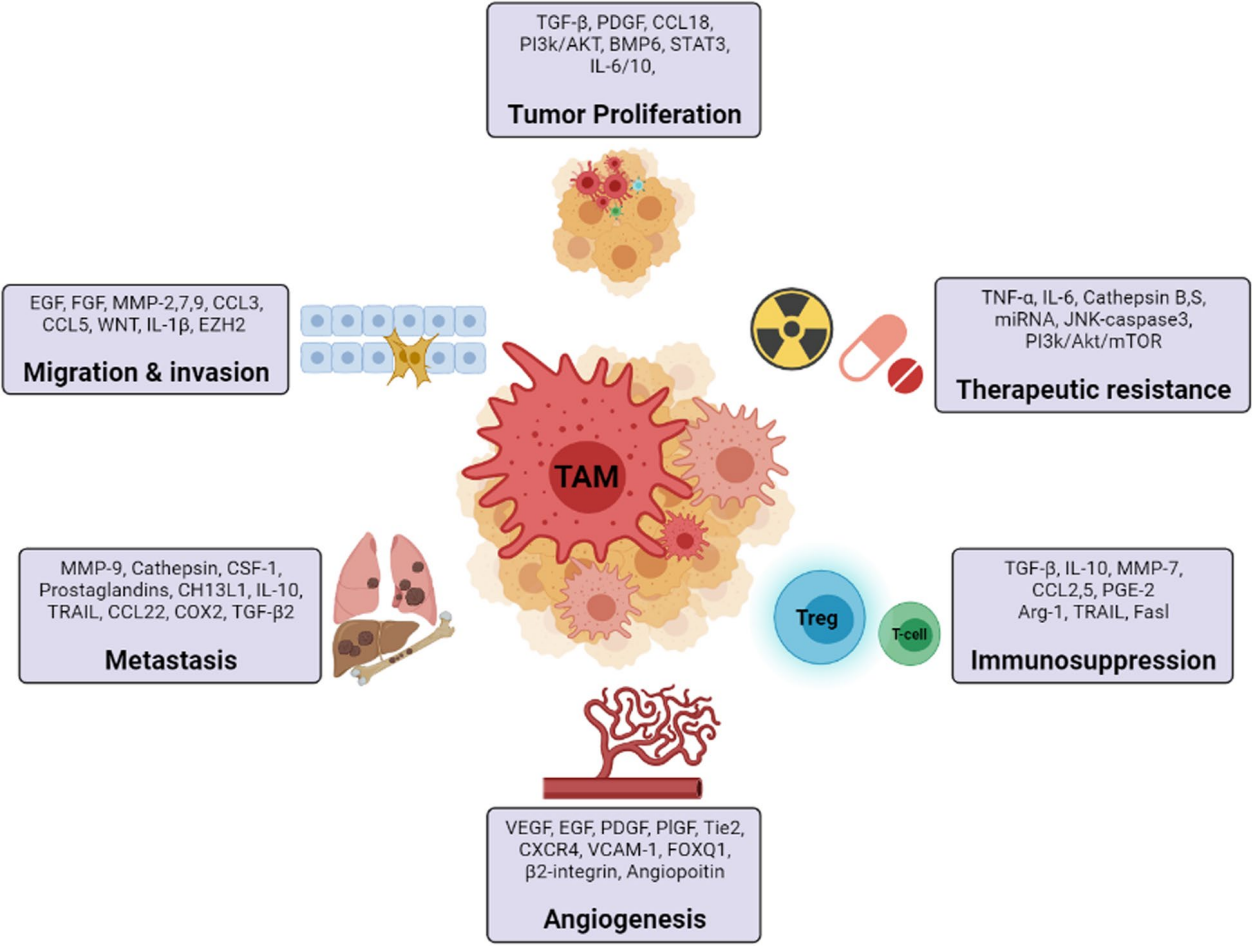
Role of TAMs in tumor development

In recent decades, TAMs have gained much attention for their magnificent ability to either restrict or facilitate tumor development. In agreement with their strong protumorigenic impact, TAM infiltration is frequently associated with poor prognosis and short survival in various cancers [49–51]. Numerous studies have reported that a higher density of M2-type macrophages is associated with increased tumor cell proliferation, vascularity, immune suppression, drug resistance, induced histological malignancy, and poor clinical prognosis [52, 53]. It should be obvious that because of their heterogeneous nature, the impact of TAMs on tumor development might fluctuate and be determined by the diversity in the TME. A schematic illustration of the effects of TAMs in tumors is shown in Fig. 1.

#### TAMs in tumor proliferation & migration

During homeostasis, not all inflammation is beneficial, and persistent inflammation promotes malignant transformation of cells and supports the tumor [54]. During tumor initiation, resident macrophages are complemented by the recruitment of monocyte-derived macrophages into the TME [5], which results in a mosaic of ontogenic diversity in TAMs that are further modified, giving rise to phenotypic and functional variability in various tumors [39, 55]. TAMs may directly induce cancer cell proliferation by releasing growth factors such as epidermal growth factor receptor (EGFR), which promote the proliferation of cancer cells [56]. Active Wnt/β-catenin signaling induced by an increased number of infiltrating macrophages can enhance the growth of tumor progenitor cells in hepatocellular carcinoma, and specific depletion of macrophages can reduce Wnt and decrease tumor growth [57]. TAMs can aid tumor progression by secreting various mediators that reshape the tumor-promoting TME. Such mediator comprises various cytokines and growth factors that induce cell proliferation and migration, proangiogenic growth factors such as vascular endothelial growth factor (VEGF), platelet-derived growth factor (PDGF), fibroblast growth factor (FGF), and transforming growth factor-β (TGF-β), NF-kB-mediated factors that protect against apoptosis, and proangiogenic growth factors [58, 59] that favor cancer cell migration and metastasis. Recently, Xia et al. reported the involvement of enhancer of zeste homolog 2 (EZH2) in tumor migration via CCL5, and interestingly, knockdown of EZH2 reduced CCL5 secretion and decreased invasion and metastasis [60]. The rapid multiplication of tumor cells causes the tumor bulk to develop quickly, which induces the necessity for nutrients and oxygen, resulting in the establishment of neoangiogenesis with elevated vascular permeability that contributes to cancer progression [61].

#### TAMs in metastasis & angiogenesis

Approximately 90% of cancer fatalities occur as a result of metastasis. TAMs behave indirectly by affecting different cell types. Type-2 cytokine-activated macrophages contribute to tissue repair and remodeling [62]. Macrophages disintegrate the extracellular matrix (ECM) by producing ECM-degrading enzymes such as MMPs, cathepsin, and many other types of proteases, which allow tumor cells to escape [63, 64]. To promote metastasis, TAMs upregulate the secretion of immunosuppressive cytokines such as IL-1ra by increasing tumor stemness [65]. Huang et al. showed that CCL5 released by TAMs enhances metastasis by activating the STAT3-β-catenin pathway, whereas silencing CCL5 in TAMs reduces tumor growth and metastasis in prostate cancer [66]. Recent research has shown that cisplatin-induced macrophages promote tumor progression and metastasis in ovarian cancer via the CCL20-CCR6 axis, which can be targeted therapeutically to reduce drug-induced metastasis in advanced-stage ovarian cancer [67]. Another recent study highlighted the importance of Wnt5a^+^ TAMs as a novel therapeutic target for combating metastasis in colorectal cancer [68]. Metastatic cells use the CCL2-CCR2 pathway to attract monocytes and differentiate them into metastasis-associated macrophages (MAMs) that support tumor cell survival and metastasis by suppressing T cells and, interestingly, ablating the recruitment of MAMs, reducing metastasis and prolonging animal survival, implying that they could be used as therapeutic targets [69, 70]. CD11b-CD18, integrins derived from M2-type exosomes, were found to accelerate the invasiveness and metastasis of cancer cells by increasing the expression of MMP-9, whereas inhibiting this axis reduced macrophage-stimulated metastasis in hepatocellular carcinoma [71]. TAMs may inhibit proapoptotic cytokines such as tumor necrosis factor-related apoptosis-inducing ligand (TRAIL) by regulating the PI3k/Akt pathways in cancer cells [72], and activation of the death receptor TRAIL-R hinders the maintenance and survival of TAMs, repairing the immune system and killing leukemic cells [73]. Exosomes derived from M2 macrophages propagate cancer by transferring miRNAs into cancer cells, including colorectal cancer and pancreatic ductal adenocarcinoma cells [74]. Moreover, TAMs also secrete several enzymes, such as iNOS, COX2 and MMPs, all of which increase angiogenesis through matrix degradation and endothelial cell invasion [75].

#### TAMs in immunosuppression

Immunosuppression is the key feature of TAM biology. In tumors, most newly differentiated macrophages originate from bone-marrow derived monocytic cells and the population of monocytic cells is composed of classical monocytes and monocytic MDSCs (M-MDSCs), which differentiate into immunosuppressive macrophages and have been identified in tumor of patients and mice [76] and play a crucial role in negative regulation of immune responses [77]. MDSCs release IL-10 to downregulate IL-12 secretion by macrophages while macrophages in turn induce MDSCs to increase IL-10 which decreases IL-6 and TNF-α in macrophages and therefore skewing the immunity towards tumor promoting type 2 response [78]. Recent studies revealed that macrophages derived from M-MDSCs expresses strong expression of S100A9, NOS2, ARG-1, SIGLEC-1 and reduced amount of HLA-DR as compared to monocytes-derived macrophages which contribute into their immunosuppressive nature [79]. Researchers recently demonstrated that tumor-infiltrating M-MDSCs downregulates the STAT3 activity through hypoxia-induced activation of CD45 phosphatase that favors their rapid differentiation into TAMs [80]. Various cytokines are also involved in the immunosuppressive function mediated by MDSC and TAM. To facilitate immune escape in melanoma, IL-1 may recruit MDSCs to promote TAM immunosuppressive programming via IL-1R-MYD88-Tet2 pathway [81]. Recently Kwak et al. demonstrated that M-MDSCs macrophages suppressed T-cell activation and could differentiate into tumor-promoting macrophages while maintaining their gene expression of their precursors and immunosuppressive activity, even in the absence of conditions associated with TME which clearly indicates that immunosuppressive function of macrophages is largely reliant on the nature of their precursors that might be the significant element in characterizing and targeting macrophage activity [79]. This study has also identified the role of S100A9 as a marker of immunosuppressive M-MDSCs-derived TAMs which also provide a potentially different approach to a selective therapeutic targeting of immunosuppressive macrophages via targeting M-MDSCs [79]. Despite the pro-tumorigenic natures of TAMs, they can eat tumor cells, induce tumor apoptosis by secreting NO, ROS, IL-12, which promote anti-tumor responses and restricts tumor development in certain circumstances [82] that suggests that immunosuppressive and immunostimulatory TAM can reside within the same tumor. Thus, selective therapeutic targeting of immunosuppressive macrophages should be designed that aim at reshaping the TAM landscape by translating TAM profile from immunosuppressive into immunostimulatory, which can be achieved by various approaches: 1) by blocking the various cell surface molecules associated with immunosuppressive TAMs such as CD206, CD204, MARCO, SIGLEC1, TREM2, CD63, PD1-PDL1 etc. [14, 55, 83], 2) by inhibiting “do not eat me” signaling to induce phagocytosis [84], 3) by disrupting the epigenetic mechanism of TAM immunosuppression such as PI3k gamma pathways, prostaglandin (PGE2) signaling or regulating the histone deacetylases etc. [85, 86]. TAM are activated by mediators secreted from tumor-infiltrating lymphocytes such as Th2, Treg cells, IL-10, TGF-β [87]. Researchers revealed the compensation between TAMs and Tregs that direct immune evasion [88]. The recruitment of Tregs cells in the TME is aided by chemokines such as CCL2, CCL3, CCL4, CCL5, CCL18, and CCL20 [89]. In colorectal cancer, TAM-derived CCL20 has been found to accelerate CCR6^+^ Tregs [90]. CCL18 enhances the recruitment of Tregs into the tumor; in contrast, blocking CCL18 inhibits Tregs migration and suppresses tumor growth [91]. Recently, Jing et al. demonstrated that CD169 (also known as SIGLEC1), a phagocytic receptor expressed by TAMs, induces immunosuppression by activating the JAK2/STAT3 pathways [92]. During tumor progression, transition of endothelial cells to mesenchymal state generate cancer-associated fibroblast which secrete HSP90α to stimulate M2 polarization and maintain immunosuppressive microenvironment [93]. CD24 on cancer cells interacts with sialic acid binding Ig-like lectin 10 (Siglec-10), expressed by TAMs, to facilitate tumor cell immune evasion in ovarian and breast cancer [94]. MHC-1 expressed by cancer cells binds with leukocyte immunoglobulins (LILRB1) on TAMs and inhibits phagocytosis, which leads to deprivation of immune surveillance; interestingly, disruption of MHC-1 or LILRB1 enhances the phagocytosis of cancer cells [95]. Complement anaphylatoxins have been found to have a role in TAM-associated T cell suppression [96]. Concurrent work by Molgora et al. demonstrated the critical role of triggering receptor expressed on myeloid cells (TREM2) in immunosuppression in various human cancers and provided strong evidence that TREM2 is a viable therapeutic target to reshape immunosuppressive TAMs to anti-tumoral effects by improving the efficacy of checkpoint blockade therapy [14]. Thus, understanding TAM regulation at the molecular level is necessary and critical in the development of tumor-targeted strategies.

#### TAMs in therapeutic resistance

Various studies have found an elevated population of TAMs after hazardous anticancer treatments. The demonstration that CSF1 suppression may cure chemoresistance of breast cancer cells in animal models was the first observation that showed TAMs might play a role in mediating chemotherapy resistance [97]. Recent studies showed the increased infiltration of TAMs after gemcitabine treatment, which decreased the therapeutic impact of drugs in pancreatic ductal adenocarcinoma (PDAC) [87, 98] and showed an enhanced therapeutic response to gemcitabine after depleting macrophages [99]. Macrophages express IL-6, TNF-alpha, and cathepsin B to activate STAT3 pathways in tumor cells, which increases the proliferation and survival of cancer cells treated with various chemotherapeutics [11]. A recent study showed aberrantly expressed glutathione S-transferase P1 (GSTP1) in TAMs from breast cancer patients, which stimulated the release of IL-6 by inactivating the NF-κB pathway [100]. Another mechanism underpinning chemoresistance has been discovered to be the epithelial to mesenchymal transition, which can be triggered by macrophages [101, 102]. High expression of CCL2 has always been found to be associated with macrophage infiltration, which was recently observed to induce resistance to tamoxifen by activating PI3K/Akt/mTOR in breast cancer [103]. TAMs have been observed to enhance glycolysis and hypoxia in non-small-cell lung cancer (NSCLC) and hinder the efficacy of PD-L1 [104]. miRNAs derived from TAMs also confer drug resistance and immune escape in various cancers [105, 106], and inhibiting the activity of miRNAs in TAMs promotes an antitumor immune response [107]. A recently published review paper indicated that TAMs contribute to drug resistance by polarizing themselves toward a protumoral phenotype and exerting antiapoptotic signals in cancer cells [108].

### Prognostic significance of TAMs

Several researchers have investigated the role of TAMs in solid cancer patients and found that higher density was significantly associated with adverse overall survival (OS) in breast, lung, liver, bladder, prostate and ovarian cancers, although disease-free survival was observed in breast and liver cancer [50, 109–111]. A meta-analysis by Zhang et al. showed that patients with a higher density of TAMs had 1.15-fold higher mortality [109]. In relation to clinicopathological parameters, high TAM density positively correlated with advanced tumor stage, higher TNM stage, severe histological grade, presence of lymphovascular invasion and metastasis in various cancers [50, 110, 112, 113]. For instance, in breast cancer, high TAM density was positively correlated with large tumor size, estrogen/progesterone receptor status, histologic grade, basal phenotype and vascular invasion [50]. Together, these studies suggested that TAMs can be used as diagnostic and prognostic markers and can also be exploited as prediction tools for the clinical outcomes of cancer patients.

### TAM-based cell therapies

One recently developed approach to encourage the TAM activation more specifically is the transduction of CARs. CARs consist of a single-chain variable fragment antibody that targets a tumor antigen fused to a transmembrane domain that attaches the antibody to the cell membrane and an intracellular domain that transmits activation and costimulatory signals [114] and is recognized as one of the greatest innovations in cancer treatment after showing excellent outcomes in blood cancer and lymphoma patients [115], although no comparable progress of its applications in solid tumors has been proven beneficial yet because T cells cannot easily penetrate and survive in the TME [116]. The TME in solid tumors recruits myeloid cells, which results in substantial infiltration of macrophages. Therefore, macrophages may be a viable substitute for T cells as CAR recipients. Macrophage-based therapies dynamically depend on TAMs, which exhibit both activating and inhibitory Fc receptors modulated to a tumor-promoting immunosuppressive phenotype and lack antigen specificity [117]. CAR for phagocytosis (CAR-Ps) is a very recent approach being explored to induce the direct phagocytosis of tumors or ECM degradation to inhibit tumor growth and progression in solid tumors. Morrissey & colleagues were the first to prove that CAR-engineered macrophages can encourage phagocytosis. They engineered a family of CAR-Ps that conduct macrophages to eat specifically targeted tumor cells. CAR-Ps contain an extracellular single-chain antibody fragment (ScFv) that recognizes CD19 and CD8 transmembrane domains present in a traditional CD19 CAR-T construct and are introduced into the murine macrophage cell line J774A by lentiviral infection. In this study, the researchers evaluated phagocytosis specificity based on the antigen recognition feature of the ScFv domain of the CAR construct and demonstrated that CAR-Ps cause antigen-coated synthetic particles and entire human cancer cells to be engulfed in a particular manner [118]. Zhang et al. engineered CAR targeting HER2 for macrophages, which consists of one variable region that binds to HER2 to increase the expression of MMPs for degradation of ECM and another intracellular region made up of CD147, which promotes the infiltration of T cells into the TME and subsequently inhibits the tumor growth in a 4 T1 murine breast tumor model [119]. Recently, CAR was shown to endow macrophages with specificity of action against tumor antigens, simultaneously with elevated antitumor functions to encourage an adaptive immune response. Inspired by the achievement of genetically engineered CAR-T cells to express antigen-specific receptors and by using the distinct effector functions of macrophages and their ability to penetrate tumors, Klichinsky et al. genetically engineered human macrophages with CAR to enhance their phagocytic capacity against tumor cells. They accomplished the transduction of anti-HER2 into primary human macrophages [CAR-macrophages (CAR-M)] using a modified replication-incompetent adenovirus. They showed that the adoptive transfer of CAR-Ms efficiently reduced tumor growth in immunodeficient mice with HER2-positive human tumors [120]. Another recent study by Zhang et al. engineered induced pluripotent stem cell (iPSC)-derived CAR-expressing macrophage cells (CAR-iMacs), which bestow antigen-dependent macrophage functions such as cytokine secretion, reprogramming toward pro/anti-inflammatory tumor states and escalated phagocytosis of tumor cells, which confers antitumor cell activities both in vitro and in vivo [121]. In another recent study, researchers developed anti-CCR7 CAR-M cells that direct macrophages toward CCR7-positive cells and remove CCR7-positive cells by screening the intracellular activation domains that trigger tumor cell cytotoxicity, which inhibit tumor growth, prevent metastasis, and induce systemic antitumor immunity, effects that collectively prolong survival [122]. The new approach based on the implementation of the ATAK platform in the evolution of two types of novel therapies will be tested in glioblastoma patients in the coming months: 1) ATAK-CAR monocytes, which combine myeloid cells with CARs against cancer cells, and 2) ATAK-primed monocytes, which act as cell vaccines and stimulate T cells against cancer cells (https://www.myeloidtx.com).

CAR-M technology is a novel therapeutic strategy to manipulate M2 macrophages toward the M1 phenotype, enhance phagocytosis, and attack cancer cells. They are genetically modified to develop an anti-inflammatory M1 profile that produces a variety of proinflammatory cytokines to stimulate antitumor changes in the TME. CAR-Ms can activate dendritic cells, recruit T cells, elevate neoantigen presentation to T cells, and contribute to the long-term adaptive immune response [120]. Regarding this, immune profiling of samples from phase-1 clinical trial patients will be critical to assess the variety of alterations induced in tumor tissue. Magnetic resonance imaging (MRI) and immune PET might be beneficial in this situation to provide additional information. The achievement of this technique offers new avenues in using engineered macrophages to exploit their potential against various tumor antigens.

### Potential treatment strategies targeting TAMs via nanomaterials

**Fig. 2:**
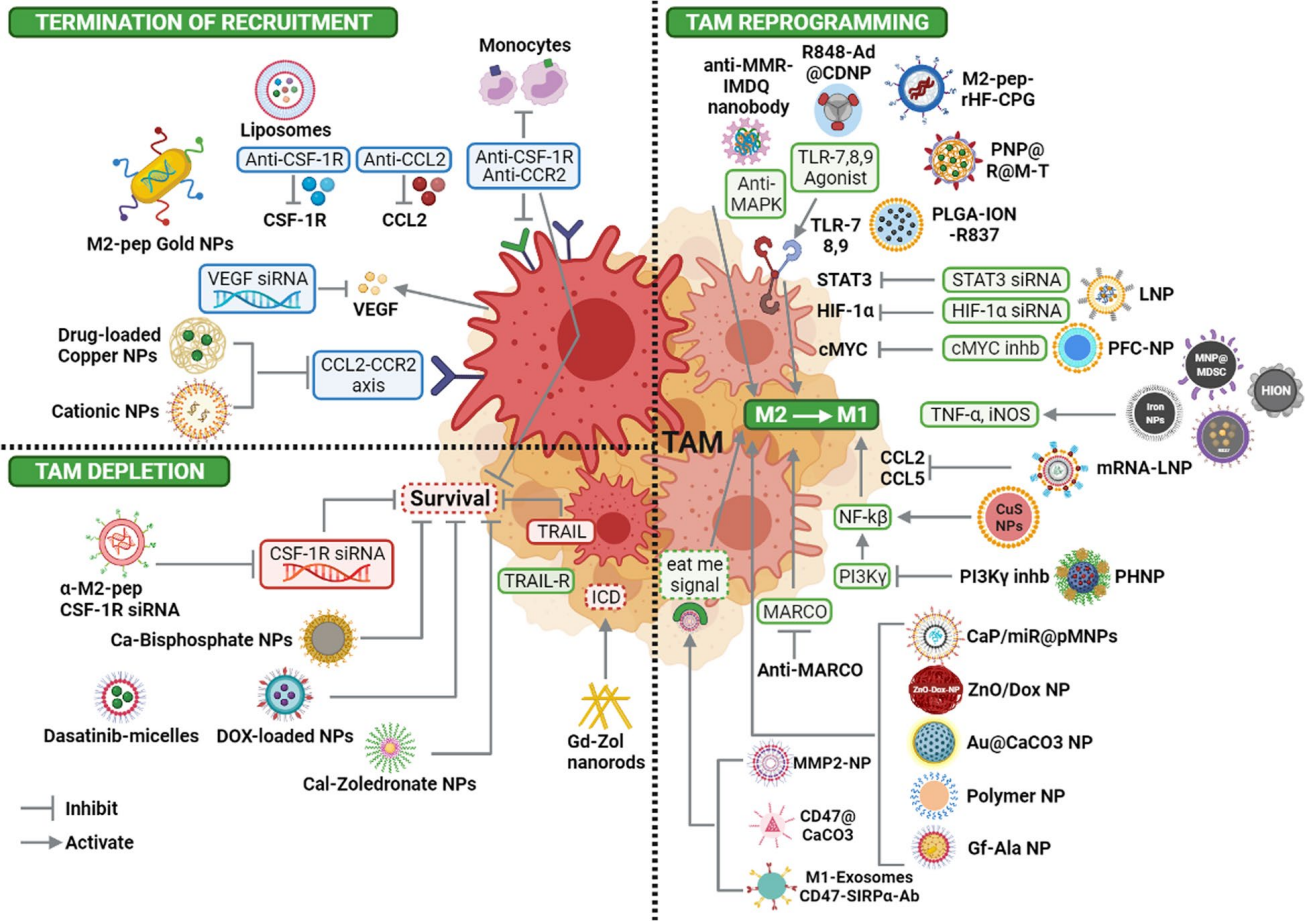
Schematic representation of TAMs-signaling pathways targeted by nanomaterial, results in inhibition of macrophages recruitment, blocking of macrophages survival and macrophage repolarization

Fundamental advancements in cancer immunology and translational immunotherapy have resulted in substantial therapeutic effects in clinical studies, including the development of advanced macrophage-based biologics, i.e., biosensors for early cancer diagnosis and CAR macrophages (CAR-Ms) for antigen-specific phagocytosis of tumors [123, 124]. Immune checkpoint inhibitors (ICIs) targeting CTLA-4, PD-L1, and PD-1 have been shown to alleviate tumor constraints of antitumor T-cell immunity in preclinical and clinical studies [125, 126]. However, their efficacy depends on the activation of immune cells, which contributes to building an immunosuppressive TME. Thus, how to obstruct these immunosuppressive cells, of which TAMs have been considered the most common cells that make up a major portion of the tumor mass, is now a matter of concern [10]. Given the functions of TAMs in tumor promotion, a variety of approaches have been developed to counteract their effects. Recently, to target TAMs, researchers have focused on the following approaches: termination of macrophage recruitment, interference of TAM survival, and reprogramming of M2-like TAMs to M1-like. The most recent advances in the specific augmentation of the anticancer immune response by targeting TAMs with nanomaterials have demonstrated great promise and potential clinical relevance (Fig. 2) and are summarized in Table 1 and will be discussed below in details.

**Table 1:** Nanomaterials developed for termination of macrophage recruitment, TAMs depletion, and TAMs repolarization

Cancers/cell lines	Strategy	Active target	Nanomaterial type	Drug	Diameter (nm)	Zeta Potential (mV)	Combination partner(s)	Outcome	Ref.
Breast cancer cell line (4 T1)	Termination of macrophage recruitment	CCR2	Cationic nanoparticle (CNP)	CNP-siCCR2	120.9 ± 12.2–128.3 ± 18.1	2.7–25.2	–	Blocking CCL2-CCR2 axis inhibited tumor growth and metastasis	[127]
Melanoma cell line (B16F10)			KLAK-MCP-1 micelles	KLAKLAK peptides	11.9 ± 2.3	7.2 ± 1.1	–	Inhibited the tumor growth via inhibiting the infiltration of TAMs and increasing the number of cytotoxic T-lymphocyte	[128]
Pancreatic ductal adenocarcinoma (THP1)			Copper nanoparticles Cu@CuO	Gemcitabine	4.9 ± 0.3	−4.8 ± 2.4	–	Induced tumor necrosis, and ultimately suppressed the tumor growth and prolong the survival in PDAC tumors	[129]
Colon cancer (SL4)		CX3CL1	7C1 nanoparticles	DC101/ anti-Ly6G antibody	–	–	7C1-Axo-siCX3CL1	Reduced the expression of CX3CL1 and prevent the recruitment of macrophages in the tumor region	[130]
Breast cancer cell line (4 T1)/ Colon cancer (CT26)/Melanoma (B16)	TAM depletion	CSF-1R	Sensitive cluster nanoparticles (^BLZ-945^SCNs/Pt)	BLZ-945	–	–	Platinum (Pt)-	Depleted TAMs, inhibited tumor growth and metastasis by increasing the infiltration of CD8^+^ cytotoxic T-cells	[131]
Melanoma cell line (B16)			M2NPs	CSF-1R siRNA	–	–	M2 macrophage binding peptide	Depleted M2-like TAMs which restored the function of T-cells and inhibit the tumor growth	[132]
Breast cancer cell line (4 T1)			Dextran-grafted-copolymer (DH@ECm)	BLZ945	~ 190	− 20.3	–	Depleted TAMs which reverse the TME with increased infiltration of CD8+ cells and inhibit the tumor growth	[133]
Bone marrow derived macrophages (BMDMs)		–	CA4 nanoparticles (A15-BLZ-NP)		163.4	−20.3		Activated anti-tumor immune response which results in improved inhibition of tumor growth	[134]
Breast cancer cell lines (4 T1, CT26, 3 T3, and RAW264.7)		Hypoxia	Calcium bisphosphonate derived nanoparticles (CaBP-PEG-NP)	–	50	−0.5	–	Deplete TAMs, normalize vascular system, reduce angiogenesis, which leads to reduction in hypoxia and inhibition of tumor growth	[135]
Sarcoma cell line (S180)		–	Lipid-coated calcium zoledronate nanoparticles (CaZol@pMNP)	–	85	Neutral	–	TAMs depletion, reduce angiogenesis and inhibit immune suppression to inhibit tumor growth	[136]
Breast cancer cell line (4 T1)		–	Mannosylated mixed micelles (DAS-MMic)	Dasatinib	21.55 ± 0.85		–	TAMs depletion, decreased angiogenesis, remodel immunosuppressive TME and inhibit tumor growth	[137]
Melanoma cell line (B16F10)		MMP-2	Phosphatidylserine nanoparticles (PS-NP)		130–230	Negative	–	TAMs depletion	[138]
Murine breast cancer cell line 4 T1			PEG liposomes (PEG-FA-Lip)	Doxorubicin	138.5 ± 6.8	−9.3 ± 0.8	–	Decreased infiltration of Treg cells to tumor sites, deplete the M2-like TAMs	[139]
Melanoma cell line (B16F10)			Hyaluronic acid-gold Nanorods (HA-AuNR/M2pep-NP)	–	42.93–64.6	–	Photothermal therapy	Eliminated M2-like TAMs, induce ICD to efficiently suppresses the tumor growth and prolongs the survival	[140]
Breast cancer cell line (4 T1)		IL-10/TGF-β/VEGF	Zoledronic acid nanorods (ZGd-NRs)	Zoledronic acid,	100–200	15.43	Radiotherapy	Improved dendritic cell maturation, promoted CD8+ T cell infiltration, and boosted the immune responses	[141]
Lung cancer (SCLC, KP1)	Repolarization of M2 to M1	–	Iron oxide nanoparticles (Ferumoxytol)	–	15–40	–	–	Prevented development of liver metastasis polarize M2-type to M1-type macrophages,	[27]
Melanoma cell lines(B16-F10)			Membrane-coated Fe3O4 nanoparticle (MNP@MDSC)	–	85–100	−18 − − 13	Photothermal therapy	Reprograming M2-like to M1-like macrophages, reduced tumor’s metabolic activity and induce immunologic cell death	[142]
Colorectal cancer cell line (CT26)/ Breast cancer cell line (4 T1)			Iron-chelated melanin-like nanoparticles Fe@PDA-PEG)		~ 150	–	–	Recruitment of M1 macrophages and attracting T-helper cells and effector cells to the tumor site to inhibit the tumor growth	[143]
Colorectal cancer cell line (MC38)		TLRs	Cyclodextrin nanoparticles (CDNP)	R848	30	0.90 ± 1.90	anti-PD-1	Shifted toward M1 phenotype and inhibit tumor growth and potentiate the efficacy of anti-tumor immune response of anti-PD-1	[144]
					39 ± 1.8	6.61 ± 1.03	–	Reprogramming of TAMs towards anti-tumor M1 phenotype	[145]
Breast cancer cell line (4 T1)			CpG- oligodeoxynucleotides ferritin Nano-cages (CpG-ODNs)	–	20.24 ± 0.29	− 11.77 ± 0.40	–	Repolarized TAMs to the M1-like in vitro and in vivo, and reduced tumor development in 4 T1 tumor-bearing animal model	[146]
Murine Melanoma B16 OVA			PLGA nanoparticles (PNP@R@M-T-NP)	R848	188	−9.7	–	Repolarize M2 to M1-type, reduce tumor size and prolong animal survival	[28]
LLC Ova cell line			Nanobodies	Imidazoquinoline	–	–	anti-PD1	Reduced the tumor growth and increased anti-tumor T-cell response by repolarizing TAMs towards pro-inflammatory phenotype	[147]
Breast cancer cell line (4 T1)		IRF5 and NF-κB signaling	Polymer magnetic nanocarrier (PLGA-ION-R837@M)	R837	166.2 ± 1.8	−19.1 ± 0.1.	–	enhanced TAMs repolarization which relieve immunosuppressive TME to activate the anti-tumor immune response	[148]
lymphoma cell lines (Raji) epidermoid carcinoma cell line (A-431) and breast cancer cell line (SKBR3)		Phagocytosis	Liposome (R848-LPs)	R848	141.9 ± 57.7	−23.9 ± 6.0	Rituximab/ Trastuzumab/ anti-EGFR mouse monoclonal antibody	Reprogram TAMs to M1-type macrophages	[149]
Breast cancer cell line (4 T1)		CD47-SIRPα	Exosome nano bioconjugates	aCD47 & aSIRPα	20	–	–	Repolarize pro-tumor M2 to anti-tumor M1 macrophages, block SIRPα & CD47, improve phagocytosis	[150]
Breast cancer cell line (4 T1)			Cell membrane-coated magnetic nanoparticles (gCM-MNs)	–	100	−19	–	Repolarization of M2-type to M1-type macrophages	[151]
Melanoma cell line (B16F10)			Calcium carbonate nanoparticles (CaCO3)	CD47 antibody	100	–	–	Activation of M1-type macrophages, inhibit local tumor recurrence and metastasis post-surgery	[152]
Breast cancer cell line (4 T1)		NF-κβ signaling pathway	Hyaluronic acid- superparamagnetic iron oxide nanoparticles (HIONs)	–	–	–	–	Reprogram M2-TAMs to antitumor M1	[153]
Breast cancer cell line (MDA-MB-231)		PI3K signaling pathway	Porous hollow iron oxide nanoparticles (PHNPs@DPA-S-S-BSA-MA@3MA)	3-methyladenine	20	−14.8 − 19.7	–	Repolarization of M2-type to M1-type macrophages and activate the immune cell population of CD8+ and CD4+ T-cells, B-cell, NK cells and Treg cells	[154]
Cervical cancer cell line (Hela) Lung carcinoma (LLC)		Lactate oxidase and glycolysis pathway	Hollow MnO2	lactate oxidase and glycolysis inhibitor	3.4	−20	Anti-PD-L1	Reduced lactic acid production and reduced population of M2-type macrophages	[155]
Hepato cellular carcinoma (JHH-7/HCA-1)		Angiogenesis	Nanocarrier (NanoMnSor)	Sorafenib	136.2 ± 1.0	−30	Anti-PD-1 antibody	Macrophage towards immunostimulatory M1 macrophages and increases the CD8+ cytotoxic T-cells in tumors	[156]
Melanoma (B16F10)		NF-κβ signaling pathway	Copper sulfide nanoparticles (CuS-NP)	–	17	–	–	Direct BMDM polarization towards anti-tumor M1-phenotype, remodels TME, prolong median survival	[157]
Hepatocellular carcinoma cell line (Hepa1–6 cells)	Repolarization of M2 to M1	–	Lipid nanoparticles (M1/SLNPs)	Sorafenib	–	–	–	Increased ratio of M1-type macrophages as compared to M2-type and inhibit the tumor growth	[158]
Hepatocellular carcinoma cells (HepG2)		NKG2D activation	Selenium nanoparticles (SeNPs)	Cytokine-induced killer cell immunotherapy	102 ± 9.6	153.4	–	Promoting M2 to anti-tumor M1 macrophages and increase the infiltration of natural killer cells to tumors	[159]
Renal cell carcinoma cell line (OS-RC-2)		STAT3/hypoxia inducible factor-1 (HIF-1α)	Lipid nanoparticles formulations (LNPs)	siRNA STAT3/ HIF-1 α	90–100	0.21 ± 0.63	–	Increased infiltration of Mφ (CD11b + cells) into the TME and increased level of M1-type macrophages,	[160]
Breast cancer cell line (PyMT-Bo1, MFI 17)/ melanoma (MDA.MB.435, MFI 27) and endothelial (HUVEC, MFI 42) cell lines		MYC pathway	Perfluorocarbon nanoparticles	MI3-PD	262	−20	αvβ3 antagonist	Decreased M2 macrophages in the TME without sparing M1-type	[161]
Breast cancer cell line (4 T1)/Melanoma cell lines (B16/F10)		CSF-1R and Src homology-region 2 (SHP-2)	Supramolecular nanoparticles	BLZ-945	143 ± 34	7.9	SHP099/ DNT206	Reprogramming of M2-type macrophages to anti-tumor M1-type	[162]
Breast cancer cell line (4 T1)		CSF-1R and MAPK pathways			190.1 ± 27	−17.1 ± 7.3	Selumetinib	Repolarize M2 macrophages to an anti-tumorigenic M1 phenotype	[163]
Hepatocellular carcinoma (Hepa1–6)/Pancreatic cancer (KPC)		CCL2/5 signaling pathways		BisCCL2/5i mRNA	100–120	–	PD-L1	TAMs polarization towards the anti-tumoral M1 phenotype and long-term survival	[164]
Breast cancer cell line (4 T1)		Macrophage Inflammatory Protein 3 Beta (MIP-3β)	Nanoparticles (3-trimethylammonium-propane (DOTAP), Methoxy poly (ethylene glycol)-poly(lactide) (MPEG-PLA), and folic acid modified poly (ethylene glycol)-poly(ε-caprolactone)	–	90	−2.1	–	Polarization of macrophages towards M1 polarization, inhibit the tumor growth and metastasis	[165]
Melanoma cell lines(B16/F10)	Repolarization of M2 to M1	–	ZnO and gold nanoparticles (AuNP@mSiO2@Dox-ZnO)	Doxorubicin	27–72	–	Photothermal treatment	Toxicity to cancer cells and contribute in immunogenic cell death, prevent tumor growth and metastasis	[166]
- (RAW 264.7 cells)		–	Gold nanoparticles encapsulated CaCO3 (Au@CaCO3-NP)	–	32	–	–	Direct repolarization of M2 macrophages towards M1-type	[167]
Osteosarcoma (MG63)/Colorectal carcinoma (HCT116)/Breast cancer (MCF7) cell lines		–	Hyaluronic acid-dexamethasone micelles (HA-DEX-DOX)	Dexmethasone & Doxorubicin	~ 252	−23 − − 26	–	M2-macrophages towards pro-inflammatory M1-type phenotype, encourage Dox-mediated apoptosis	[168]
Melanoma cell line (B16)		–	PLGA nanoparticles	Baicalin	97.2–123.6	−43.1 ± 0.4/ -17.8 ± 0.3	Hgp	Transformation of M2-like TAMs to M1-like, suppress tumor angiogenesis, inhibited metastasis, stimulate NK cell infiltration	[169]
Colorectal cancer (CT26-FL3), Pancreatic (PDAC) and breast cancer (4 T1) cell lines		–	Lipid calcium phosphate nanoparticles (LCP)	–	189.5	−6.82	PD-L1	Inhibited the metastasis and shifted the immunosuppressive TME towards immunostimulatory stage with better cytotoxic T-cell infiltration, which results in prolong animal survival	[170]
Breast cancer cell line (4 T1)		–	Polymer nanoparticles (P-NPs)	–	45	−25	Photodynamic Therapy	Shifts macrophages towards anti-tumorous phenotype, reverse TME and inhibit the tumor growth	[171]
Breast cancer cell line (4 T1) or human pulmonary carcinoma cell line (A549)		NF-κB and IRF5 pathways	Gadofullerene nanoparticles (Gd@C82)	–	~ 55	−37.7 ± − 0.3	PD-L1	Reprogram M2 to M1, induce infiltration of cytotoxic T-lymphocytes and inhibit the tumor growth, promotes efficacy of PD-L1	[172]
Desmoplastic Melanoma (BPD6).			Liposomes	Hydralazine Doxorubicin	88 ± 4	−1.8	–	Repolarize the TAMs by normalizing tumor blood vessels, effectively inhibit melanoma growth	[173]
Melanoma cell line (A375)		Mitochondrial-mediated apoptosis pathway	Tellurium Nano stars (GTE-RGD)	–	170	19.9 to + 19.6	PD-1 & Radiotherapy	Increase M1 macrophages, potentiated radiotherapy, eradicate tumor, enhance cytotoxic T-lymphocytes	[174]
Breast cancer (MCF-7) Renal cell carcinoma (A498)/Lung adenocarcinoma cell line (A549)		NF-κB and STAT3 pathways	TCCP-loaded mPEG-PLGA polymeric nanoparticles	–	80 ± 1.5	−11.8 ± − 0.8	Photodynamic Therapy	Enhance polarization to M1 macrophages, induced immunogenic cell death, increase anti-tumor immunity of NK cells	[175]

#### Termination of macrophage recruitment

**Fig. 3:**
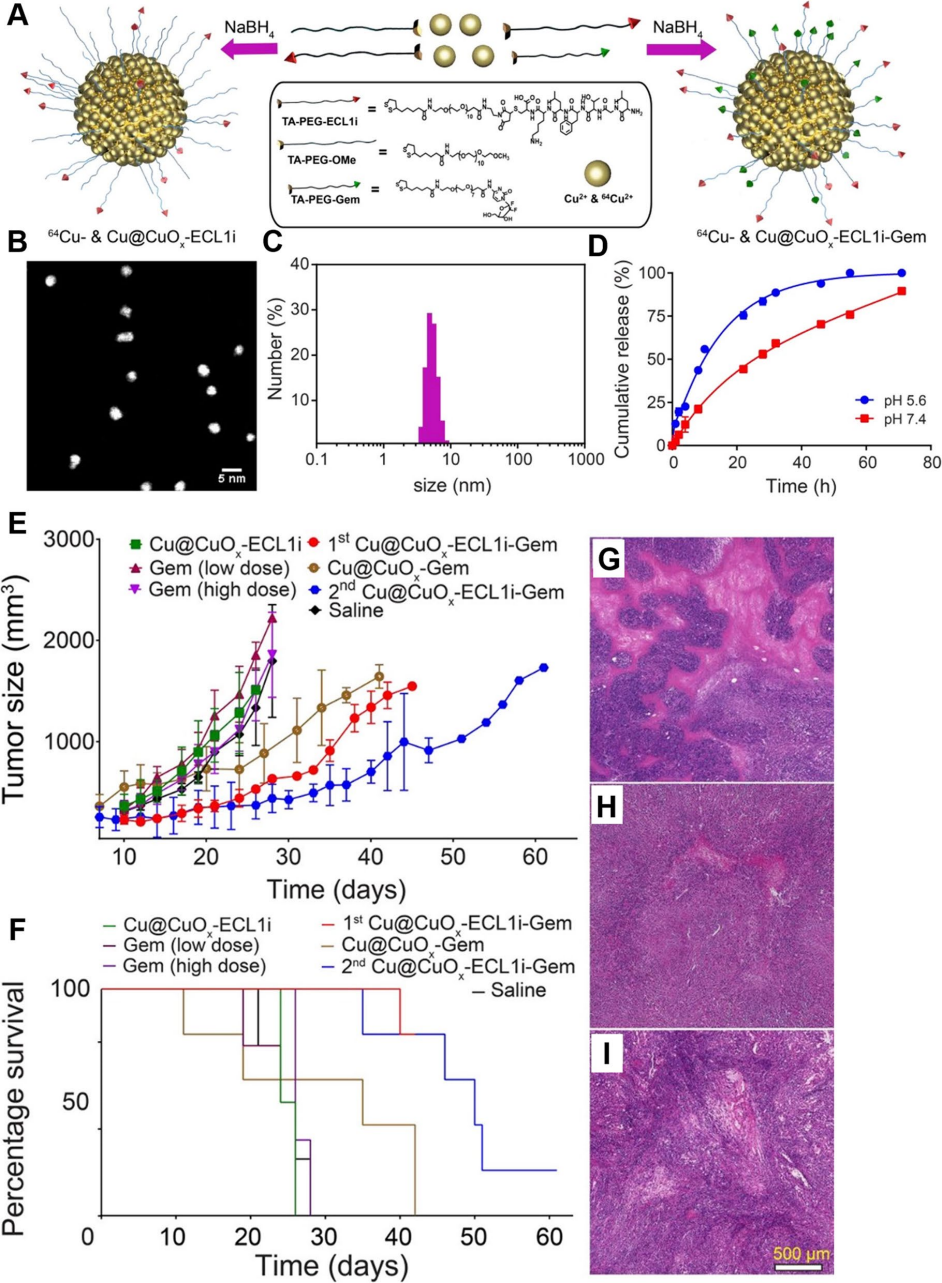
**A** Schematic diagram of the synthesis of Cu@CuO*x*-ECL1i, Cu@CuO*x*-ECL1i-Gem, and 64Cu-radiolabeled nanoparticles. **B** STEM of Cu@CuO*x*-ECL1i. **C** Number-average hydrodynamic diameter of Cu@CuO*x*-ECL1i. **D** In vitro TA-PEG-Gem release profiles of Cu@CuO*x*-ECL1i-Gem under physiological and acidic conditions. **E** Tumor growth (**F**) and mouse survival, curves of KI-implanted mice after being treated with Cu@CuO*x*-ECL1i-Gem, Cu@CuO*x*-ECL1i, Cu@CuO*x*-Gem, gemcitabine (7 mg/kg body weight, IV), gemcitabine (100 mg/kg body weight, IP), and saline. First treatment with Cu@CuO*x*-ECL1i-Gem started at 10 days’ post-tumor implantation. The Cu@CuO*x*-Gem and second treatment began at 7 days’ post-tumor implantation. **G** H&E staining of the tumor slices from mice treated with Cu@CuO*x*-ECL1i-Gem, **H** gemcitabine (100 mg/kg body weight), and (**I**) saline [129]

Multiple studies have demonstrated the prominent role of chemokine ligands secreted by the TME in regulating TAMs and progenitor cells [176]. Therefore, macrophage-recruiting chemokines (CCL2, CCL3, CCL4 & CCL5), CSF-1, and VEGF are possible therapeutic targets that can stop malignant tumors from spreading by interrupting prometastatic TAMs [177, 178]. To block the CCL2-CCR2 axis, researchers designed siRNA-CCR2-encapsulated cationic nanoparticles (CNP-siCCR2) to suppress the expression of CCR2 in monocytes by blocking the CCL2-CCR2 axis and reshaping the TME to inhibit tumor growth and metastasis [127]. Jung et al. engineered 7C1 nanoparticles loaded with CX3CL1 on 7C1, which successfully reduced the expression of CX3CL1 and prevented the recruitment of macrophages toward the tumor region [130]. Recently, KLAK-MCP-1 micelles containing a CCR2-targeting peptide sequence and apoptotic KLAK peptide to induce apoptosis were synthesized to interrupt the MCP-1/CCR2 axis, which successfully inhibited tumor growth in B16F10 melanoma by inhibiting the infiltration of TAMs into the tumor and elevating cytotoxic T lymphocytes [128]. In another recent study, the researcher engineered CCR2-targeting ultrasmall copper nanoparticles (Cu@CuO_x_) as a nanovehicle loaded with gemcitabine for PET-guided drug delivery into pancreatic ductal adenocarcinoma tumors. These nanoparticles specifically target CCR2 on monocytes/macrophages and successfully inhibit the recruitment of TAMs to the tumor, which synergizes with the therapeutic effect of gemcitabine, leads to tumor necrosis, and ultimately suppresses tumor growth and prolongs the survival of PDAC tumors under imaging-guided therapy (Fig. 3). Additionally, these ultrasmall nanoparticles showed rapid clearance from the body to reduce toxicity [129].

#### TAM depletion

Suppressing one of the various chemoattractants is too simple to entirely prevent macrophage recruitment to the tumor. Regardless, once TAMs have infiltrated the tumor, they can be removed by various methods. Given the importance of CSF-1R in macrophages, numerous clinical medicines targeting CSF-1R have been discovered, such as BLZ945, PLX3397, PLX7486 and PLX7486 [179, 180]. Researchers have shown that CSF-1R inhibitor-loaded nanoparticles efficiently deplete TAMs and inhibit tumor growth and metastasis [131]. Researchers also designed dual-targeting nanoparticles (M2NPs), regulated by α-peptide coupled with M2-pep (M2 macrophage binding peptide) and loaded with anti-CSF-1R-siRNA, to precisely obstruct the survival signal of M2-type TAMs, which restored T cells and inhibited tumor growth in melanoma tumors [132]. Recently, Wei et al. developed FXIIIa substrate peptide A15-decorated BLZ945 nanoparticles (A15-BLZ-NPs) to selectively target M2-like TAMs and to escalate the efficacy of the antitumor effects of combretastatin A4 nanoparticles (CA4-NPs). Here, A15-BLZ-NP selectively targeted CA4-NP-treated tumors with elevated M2-like TAMs, where they release BLZ945 to deplete M2-like TAMs specifically. CA4-NP improves the delivery of A15-BLZ-NP to tumors, and A15-BLZ-NP specifically targets M2-like TAMs, which collectively leads to remodeled and activated antitumor immune responses that inhibit tumor growth [134]. Currently, various erythrocyte membrane-coated nanoformulations have gained much interest due to their improved immune camouflage characteristics in antitumor research [181]. Recently, researchers designed novel erythrocyte-cancer cell membrane-coated histidine copolymer micelles to deliver BLZ945 that holds immune camouflage capability to prolong circulation time and specifically deplete TAMs, which increases the infiltration of CD8^+^ cells and inhibits tumor growth [133]. Previous studies reported that bisphosphates can specifically obstruct macrophage survival and benefit nanotechnology [182]. Tian et al. synthesized PEGylated calcium bisphosphate (CaBP-PEG) nanoparticles with CaCl2 and bisphosphate that deplete TAMs, normalize the vascular system, and reduce angiogenesis, leading to a reduction in hypoxia and inhibition of tumor growth in breast cancer [135].

Surface markers of macrophages, such as CD206, can also be used as therapeutic targets [183]. Based on this concept, Zang et al. developed nanotherapeutics of lipid-coated calcium zoledronate nanoparticles (CaZol@pMNPs) enclosing conjugated mannose and covered with an extracellular pH-sensitive material that showed increased cellular internalization and detachment of PEG in low pH-TME, uncovered mannose to encourage delivery of zoledronate for TAM depletion, reduced angiogenesis and inhibited immune suppression to reduce tumor growth [136]. Recently, Zhang et al. synthesized mannosylated mixed micelles loaded with dasatinib (DAS-MMic) that selectively deplete TAMs, reduce the proportion of M2 macrophages, decrease angiogenesis, remodel the immunosuppressive TME, and inhibit tumor growth in a 4 T1 tumor-bearing mouse model [137].

Phosphatidylserine (PS) on the external surface of the cell membrane indicates an “eat me” signal for phagocytic clearance by macrophages and has been used as a targeting ligand in nanoparticles [184]. Knowing that MMP-2 is overexpressed by cancer cells [185] and PS-induced phagocytosis of apoptotic cells, MMP-2-sensitive nanoparticles covered with PS loaded with dasatinib were developed recently by Liu. In this study, PS externalization on the surface of nanoparticles was regulated by MMP-2 secreted by tumor cells, which rendered an “eat me” signal to TAMs for tumor-specific phagocytosis. Here, the author demonstrates the considerable accuracy and efficiency of these nanoparticles in TAM targeting and drug delivery in various biological and tumor-bearing mouse models [138]. Deng et al. also synthesized MMP-2-responsive, folate-modified Dox-loaded liposomes (PEG-FA-Lip) to target both M2-like TAMs and 4 T1 breast cancer cells. PEG-FA-Lip induces immunogenic cell death (ICD) at the tumor region, decreases the infiltration of Treg cells to tumor sites, depletes M2-like TAMs and significantly suppresses the tumor volume in a breast cancer model [139]. Recently, Tian et al. developed photoimmunotherapy nanoparticles, denoted HA-AuNR/M-M2pep, made up of hyaluronic acid-modified gold nanorods that have been surface-modified with MMP2-responsive M2-pep fusion peptides. HA-AuNR/M-M2pep showed better accumulation at tumor sites and discharged M2-pep in the TME with high MMP2 expression to specifically eliminate M2-like TAMs, which induced ICD to efficiently suppress tumor growth and prolong the survival of melanoma-bearing animals [140].

**Fig. 4:**
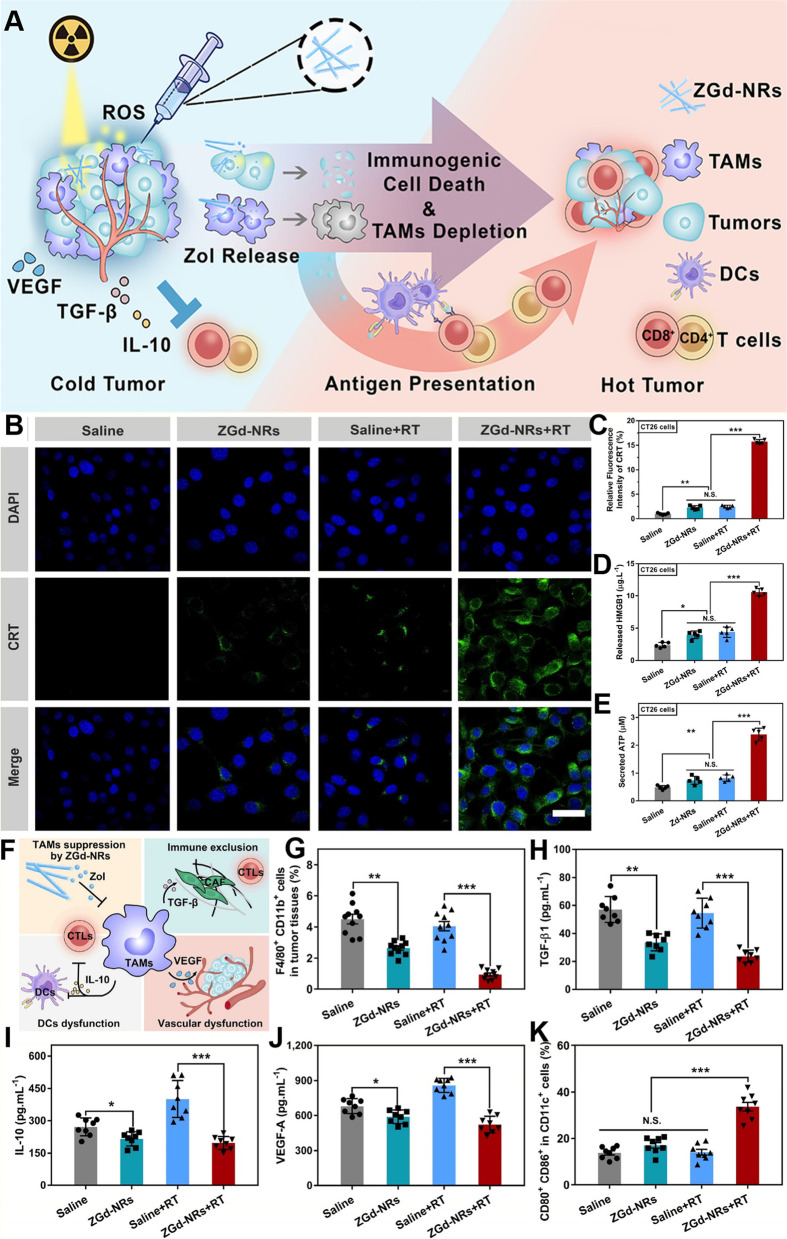
**A** Mechanism of ZGd-NR-sensitized radiation for ICD induction and TAM depletion to synergistically boost antitumor immunity. **B**-**K** Immunogenic cell death induction and immunosuppressive tumor microenvironment reprogramming. **B** Immunofluorescence of CT26 cells stained with anti-CRT antibody, scale bar = 20 μm. **C** Quantification of relative CRT mean fluorescence intensity (*n* = 5 biologically independent cells). **D** Detection of cytoplasmic HMGB1 by ELISA kit (*n* = 5 biologically independent cells). **E** Detection of ATP secretion by luciferin-based ATP assay kit (*n* = 5 biologically independent cells). **F** Regulation of tumor immunity by TAMs. **G** Flow cytometry analysis of TAMs (F4/80+ and CD11b+) in tumor tissues (*n* = 10 biologically independent animals). **H** Quantification of the levels of TGF-β1 (**I**), IL-10 (**J**), and VEGF-A in tumor tissues by ELISA kit, the tumor tissues were harvested 48 h after radiotherapy (0 or 6 Gy × 1, *n* = 8 biologically independent animals). **K** Flow cytometry analysis of DCs maturation (CD80+ and CD86+ gated on CD11c+) in tumor-draining lymph nodes; the TDLNs were harvested 5 days after radiotherapy (0 or 6 Gy × 1, *n* = 8). All data were shown as mean ± SD. N.S. represented non-significance, *p < 0.05, **p < 0.01, ***p < 0.001 [141]

It has been determined that radiation therapy can ionize DNA or produce reactive oxygen species (ROS), which release tumor-associated antigens (TAAs) and induce the production of damage-associated molecular patterns inside the TME, which cause ICD [186, 187]. Recently, Huang et al. constructed self-assembled dual-functional coordination nanorods based on zoledronic acid and gadolinium (ZGd-NRs) that can effectively deposit X-rays and generate a large amount of hydroxyl radicals to stimulate ICD (Fig. 4). Eventually, ZGd-NRs can specifically eliminate TAMs, reprogram the immunosuppressive TME by inhibiting TGF-β, IL-10 and VEGF, enhance the infiltration of CD8^+^ T cells and significantly potentiate the immune response to anti-PD-L1 treatment in primary, distant and metastatic tumors [141].

#### Reprogramming of M2 to M1

Although macrophage depletion may have some benefits in the initial stage of disease, scientists have previously ignored the advantageous effects of M1 macrophages. Instead, researchers should focus on the potential negative consequences of systemic macrophage depletion, such as increased susceptibility to infection and impairment of homeostatic macrophage functions in heathy tissue. The primary goal of immunotherapy is to reverse the immunosuppressive TME to antitumor, although the biggest obstruction is that it is quite difficult for conventional drugs to target only M2 macrophages. As described above, the unique ability of macrophages to modify their phenotype in response to external changes has been recognized for a long time. Therefore, to achieve the benefits of M1 macrophages, reprogramming tumor-promoting M2-type macrophages to tumoricidal M1 macrophages could be a more beneficial approach in tumor eradication than total macrophage depletion.

Currently, iron oxide nanoparticles have attracted much interest due to their broad range of biomedical applications in cancer theranostics [188]. Zanganeh et al. demonstrated that ferumoxytol induces macrophage polarization toward a proinflammatory phenotype and elicits strong antitumor effects through Fenton’s reaction in adenocarcinoma [27]. However, these effects are not sufficient because tumor cells disguise their immunogenicity, and most nanoparticles can be phagocytosed by phagocytes and may never reach their target. To solve this issue, Yu et al. designed a myeloid-derived suppressor cell (MDSC) membrane to coat magnetic Fe_3_O_4_ nanoparticles (MNPs@MDSCs) for enhanced antitumor activity. As a major regulator of immune responses in cancer, the MDSC membrane allows immune escape, easily accumulates in the TME, is capable of reprogramming M2-like to M1-like macrophages and induces ICD [142]. Various studies have shown that photothermal therapy (PTT) kills tumor cells, induces the production of TAAs, rebuilds the TME, and activates the specific T-cell immune response [189]. Researchers utilize the advantages of PTT in combination with iron-chelated melanin-like nanoparticles (Fe@PDA-PEG), which allows the recruitment of M1 macrophages as professional antigen-presenting cells to present TAAs, which results in attracting T-helper cells and effector cells to the tumor site to inhibit tumor growth [143].

**Fig. 5:**
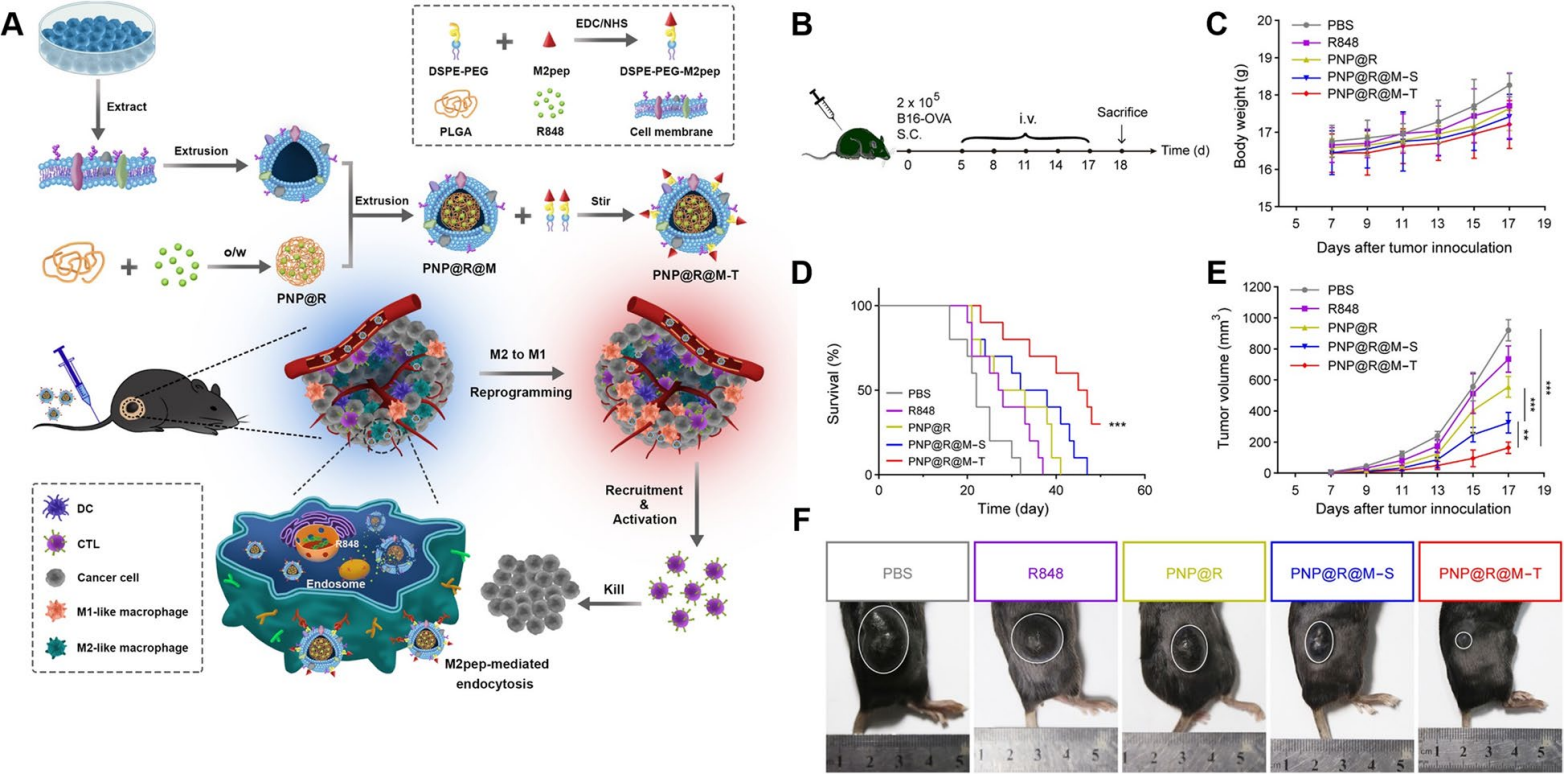
**A** Schematic illustration of PNP@R@M-T developed for efficient and selective reprogramming of M2-like macrophages and enhanced cancer immunotherapy via M2pep-mediated endocytosis. **B-F** Inhibitory effects of PNP@R@M-T on tumor growth in vivo. **B** Schematic illustration of induction and treatment of B16-OVA tumors in C57BL/6 mice. **C** Body weights of mice treated with PBS, R848, PNP@R, PNP@R@M-S, and PNP@R@M-T. **D** The survival rate was analyzed by the log-rank test (*n* = 10 mice). **E** B16-OVA tumor growth. *n* = 6 mice. **F** Macroscopic images of tumors taken 18 days after the initiation of treatment. Representative images from 6 mice per group are shown [28]

**Fig. 6:**
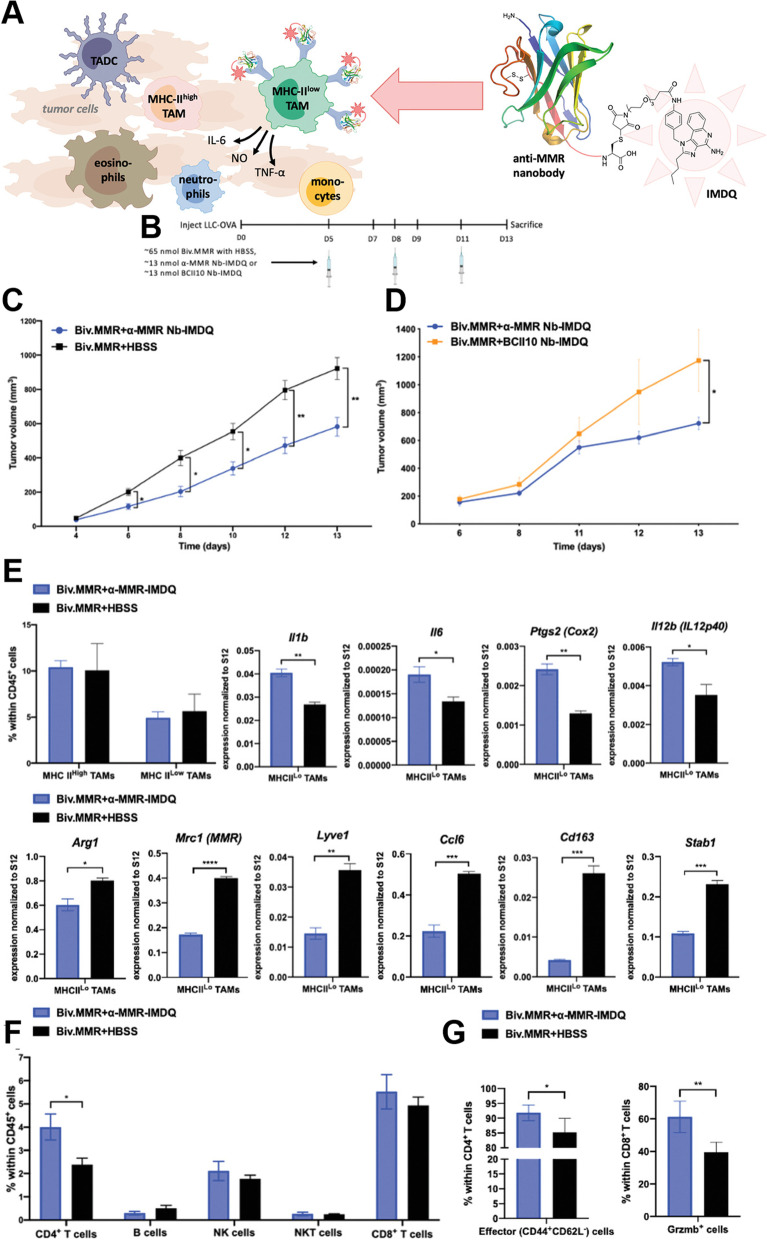
**A** A well-defined protein-drug conjugate of anti-MMR nanobody with TLR 7/8 agonist IMDQ. The anti-MMR Nb-IMDQ conjugate allows triggering of TLR7/8 specifically of MMR high macrophages, with aim to repolarize these cells into a pro-inflammatory anti-tumoral state, resulting in reduced tumor growth. **B-G** Anti (α)-MMR Nb-IMDQ therapy delays tumor progression and reprograms TAMs to more M1 phenotype. **B** LLC-OVA-bearing C57BL/6 mice were injected on day 5, 8, and 11 after cancer cell inoculation with appropriate treatment and mice were sacrificed on day 13. **C** LLC-OVA bearing mice received α-MMR Nb-IMDQ or HBSS, co-injected with fivefold molar excess of bivalent α-MMR Nb (Biv.MMR) and tumor volumes were measured on day 4, 6, 8, 10, 12, and 13 after cancer cell inoculation. **D** LLC-OVA bearing mice received α-MMR Nb-IMDQ or BCII10 Nb-IMDQ, co-injected with fivefold molar excess of Biv.MMR, tumor volumes were measured on day 6, 8, 11, 12, and 13 after cancer cell inoculation. *p*-values are calculated using a two-way ANOVA and significant differences are marked by *: *p* ≤ 0.05. **E** The percentage of MHC-II high and MHC-II low TAMs within hematopoietic (CD45+) cells of LLC-OVA tumors is shown as mean ± SEM of *n* = 4. MHC-II low TAMs were sorted from pools of tumor cell suspensions of each individual experimental group and qRT-PCR analysis was performed for technical triplicates to quantify expression of several M1 and M2-associated genes normalized to ribosomal protein S12 expression. **F** Percentage of CD4+ T cells, B cells, NK cells, NKT cells, and CD8+ T cells within the hematopoietic (CD45+) cells is shown as mean ± SEM of *n* = 4, *p* ≤ 0.05. **G** Percentage of effector (CD44 + CD62L−) cells within CD4+ T cells and Gzmb+ cells within CD8+ T cells is shown as mean ± SEM. p ≤ 0.05; **: *p* ≤ 0.01 [147]

Toll-like receptor (TLR)-agonists have been recognized as agents to polarize M2-like TAMs to M1-like and are under clinical trial as anticancer agents to determine their suitability for nanomedicine [190]. Rodell et al. designed β-cyclodextrin nanoparticles (CDNPs) that encapsulate R848 (an agonist of TLR-7/8), a vigorous driver of the M1 phenotype. The rapid uptake of these nanoparticles effectively improved drug delivery to TAMs, shifted them toward the tumoricidal M1 phenotype, inhibited tumor growth in colorectal cancer, and potentiated the efficacy of the antitumor immune response of anti-PD-1 [144]. Recently, the same authors showed the efficiency of the same nanoparticles (CDNP-R848) in a murine MC38 cancer model, in which they demonstrated the reprogramming of TAMs toward the antitumor M1 phenotype [145]. Undoubtedly, TLR agonists can activate proinflammatory macrophages, but their use in vivo is limited due to a lack of efficient delivery methods, and they can be cleared easily from the circulation. Researchers believe that nanoparticles can deliver TLR agonists to tumors. Recently, Shan et al. designed an M2-targeted nanocarrier system by encapsulating CpG-ODN in ferritin nanocages, surface functionalized with M2-targeting M2 peptide, denoted as M2pep-rHF-CpG. Following intravenous treatment, M2peprHF-CpG nanoparticles effectively repolarized M1-like TAMs in vitro and in vivo and reduced tumor development in a 4 T1 tumor-bearing animal model [146]. To increase the efficacy and specificity of macrophages, Zhang et al. recently prepared TLR agonist-loaded nanoparticles to modulate the TME. Here, they encapsulated R848 into polylactic-coglycolic acid (PLGA) and then covered the B16-OVA cancer cell membrane (to avoid being removed by the reticuloendothelial system), which was further modified with M2-pep and designated PNP@R@M-T (Fig. 5). The author showed that PNP@R@M-T specifically delivers the drug to M2 macrophages and dramatically repolarizes them toward the M1 type, activates the antitumor immune response, reduces tumor size and prolongs animal survival [28]. Recently, researchers have also focused on strategies to utilize the potential of the Fenton reaction in combination with TLR agonists to potentiate immunotherapies. In one recent study by Liu et al., the author designed a cell membrane-coated nanocarrier system, PLGA-ION-R837@M, consisting of magnetic Fe_3_O_4_ and R837, coated with LPS-treated macrophage membranes to target TAMs. The synergistic interaction between Fe_3_O_4_ and R837 activates interferon regulatory factor 5 (IRF5) and the NF-*κ*B signaling pathway to enhance TAM repolarization, which relieves the immunosuppressive TME to activate the antitumor immune response [148]. Recently, Bolli et al. designed a strategy to couple the TLR7/8 agonist imidazoquinolinone to single-chain antibody fragments (anti-MMR Nb-IMDQ), target mannose receptor (MMR) on macrophages in a site-specific and quantifiable manner and repolarize protumoral TAMs into an antitumoral type. The anti-MMR Nb-IMDQ conjugates resulted in efficient drug delivery to TAMs high in MMR expression and significantly decreased tumor growth, aligned with an increased antitumor T-cell response by repolarizing TAMs toward a proinflammatory phenotype (Fig. 6) [147]. TAMs were found to be required for antibody-dependent cell phagocytosis, which has been considered a key mechanism for antibody cancer therapy. A recent study demonstrated the potency of R848-encapsulated liposomes (R848-LPs) to accumulate quickly in tumor sites, including TAMs, reprogram TAMs to M1-type macrophages and potentiate antibody-dependent phagocytosis in lymphoma cell lines [149].

**Fig. 7:**
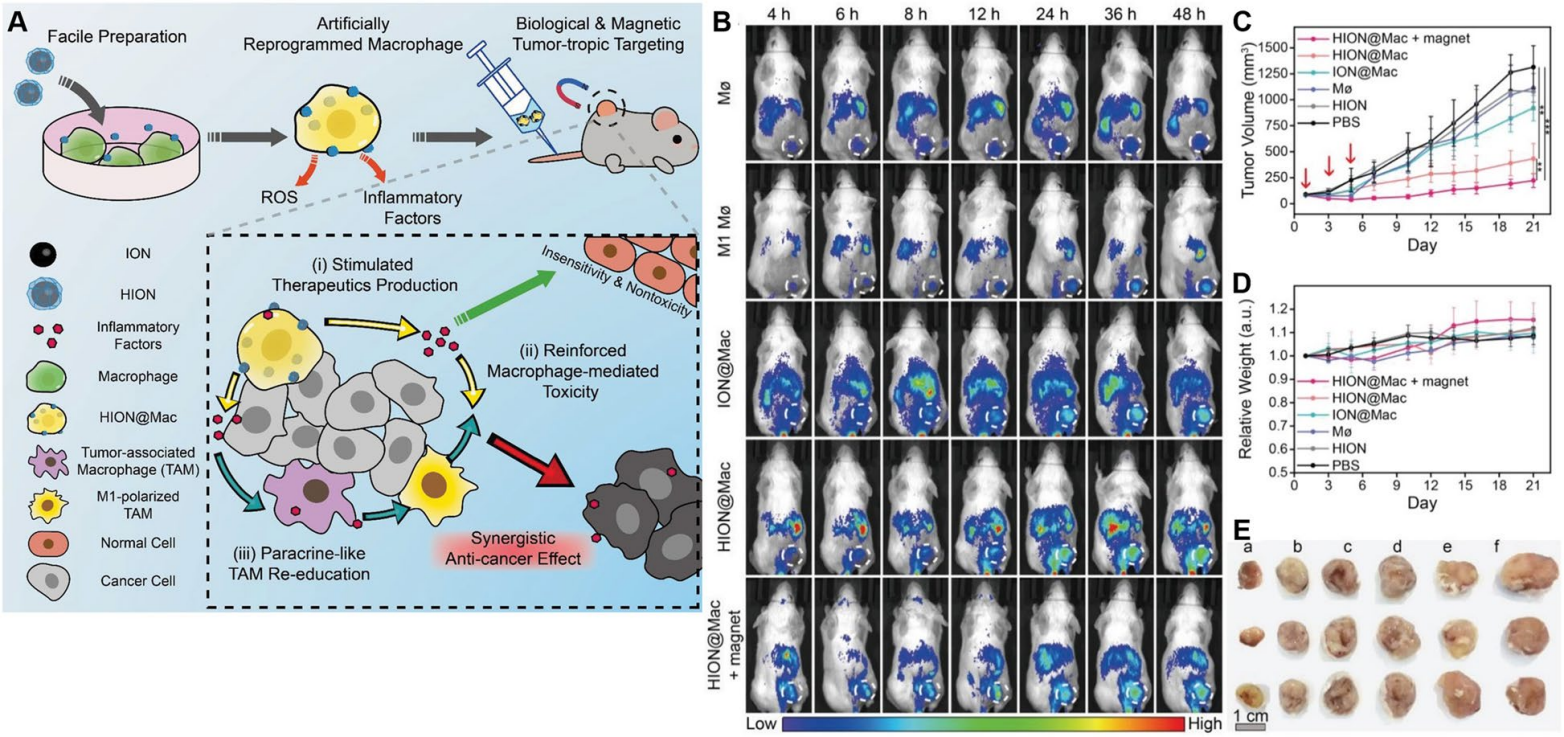
**A** Schematic illustration depicting that the artificially reprogrammed HION@Macs target tumors through active chemotaxis and magnet guidance, produce inflammatory factors (such as TNF-α, NO and ROS) to suppress tumor, re-educate in situ M2 macrophages into pro-inflammatory M1 phenotype for synergistic cancer-specific therapy. **B-E** In vivo tumor targeting and anticancer effect of HION@Macs in BALB/c mice bearing subcutaneously inoculated 4 T1 breast tumor. **B** Representing IVIS images depicting bio distribution of Møs, M1 Møs, ION@Macs, HION@Macs, HION@Macs plus magnet guidance. The tumor site was designated by white dotted circle. **C** Tumor growth profiles recorded during 21 days. Tumor bearing BALB/c mice received a total of three injections on the 1st, 3rd, and 5th day (designated by red arrow) since tumor volume reached ≈80 mm3. The asterisks indicate the difference between the HION@Macs + magnet group, the HION@Macs group, and the PBS group. **: *p* < 0.01; ***: *p* < 0.001. **D** Relative body weight of mice from different groups after treatments. **E** Representative image of tumor tissues harvested from different groups on the 21st day. Group a) HION@Macs + magnet; b) HION@Macs; c) ION@Macs; d) Mø; e) HION; f) PBS (Scale bar: 1 cm). Error bars represent mean ± S.D. (*n* = 6) [153]

Exosomes have recently shown significant promise in cancer treatment. Recently, Nie et al. designed pH-responsive M1 exosome nanobioconjugates for cancer treatment in which dibenzocyclooctyne-modified antibodies against CD47 and SIRPα were conjugated with azide-modified M1 exosomes linked with pH-sensitive benzoic-imine bonds. On intravenous administration, they specifically recognize CD47 on the surface of tumor cells by aCD47. The acidic environment of the tumor stimulates the cleavage of the benzoimide bond of nanobiological coupling and discharges aSIRPα and aCD47 on macrophages, which abolishes the “do not eat me” signal and stimulates the phagocytosis of tumor cells. Simultaneously, M1-derived exosomes effectively repolarize macrophages from the protumor M2 type to the antitumor M1 type [150]. Recently, researchers used a gene editing technique to construct genetically modified cell membrane-coated magnetic nanoparticles (gCM-MNs) to encourage macrophage membrane surface overexpression of the SIRPα protein, which blocks the CD47-SIRPα pathway. The magnetic core encourages the repolarization of M2-type TAMs to M1, synergistically triggers a potent macrophage immune response and suppresses tumor growth in breast cancer [151]. Chen et al. developed a postsurgical immunotherapeutic fibrin gel (aCD47@CaCO_3_) using calcium carbonate nanoparticles preloaded with CD47 antibody that gradually dissolve and release aCD47 in tumors in a controlled manner, which encourages the activation of M1-type macrophages and induces the phagocytosis of cancer cells by macrophages, boosts the antitumor immune response and inhibits local tumor recurrence and metastasis postsurgery [152]. Researchers have also investigated a facile way to develop artificially reprogrammed macrophages as live cell therapeutics. They prepared tremendously activated macrophages (HION@Mac) that exhibited outstanding advantages, including activation of the NF-κB signaling pathway and stimulation of macrophages to continuously produce TNF-α and ROS, which induce therapeutic effects against tumors. Interestingly, HION-reprogrammed TAMs resisted the immunosuppressive TME and simultaneously reprogrammed M2-TAMs to antitumor M1 macrophages in a paracrine-like manner (Fig. 7). This study paves the door for cell-type immune therapeutics to enter clinical practice [153].

The PTEN/PI3K γ/mTOR signaling pathways have been demonstrated to regulate the TME by repolarizing macrophages and promoting immune suppression during cancer development [191]. Therefore, scientists synthesized porous hollow iron oxide nanoparticles loaded with a PI3K γ inhibitor (3-methyladenine) and blocked them with bovine serum albumin (BSA). Furthermore, their surface was modified by carbonylated mannose (PHNPs@DPA-S-S-BSA-MA@3-MA) to effectively target TAMs only. They upregulated NF-kB p65 by reducing the PI3Kγ protein in macrophages and tumor cells, which efficiently polarized macrophages toward proinflammatory macrophages and activated the immune response by increasing the population of CD8^+^ and CD4^+^ T cells, B cells, NK cells and Treg cells, collectively inhibiting tumor growth and reshaping the immunosuppressive TME [154].

**Fig. 8:**
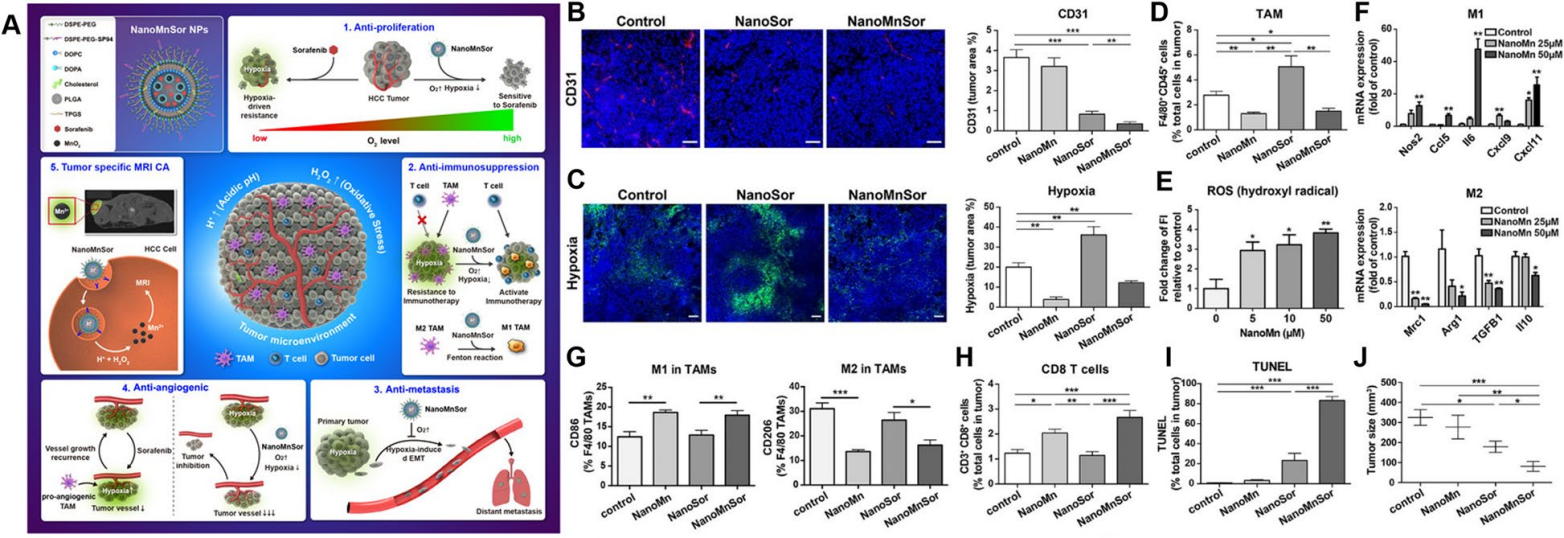
**A** Schematic representation of mechanism by which NanoMnSor can serve as theranostic anticancer agent. Oxygen generated from NanoMnSor alleviates tumor hypoxia and modulates TME. (1) NanoMnSor treatment overcomes hypoxia-driven resistance to sorafenib and reduces cell proliferation in HCC. (2) NanoMnSor ameliorates immunosuppressive TME by reducing hypoxia-induced tumor infiltration of TAMs, promoting macrophage polarization toward immunostimulatory M1, increasing CD8+ cells, leading to improving efficacy of anti-PD-1 and whole-cell cancer vaccine. (3) NanoMnSor suppresses metastasis in HCC by attenuating hypoxia induced EMT. (4) NanoMnSor treatment enhances antiangiogenic effect of sorafenib via hypoxia alleviation. (5) NanoMnSor potentially serves as CA for tumor imaging because of acidic and redox-active TME-induced decomposition of MnO2 core into Mn2+ ions that enhances tumor contrast in T1-weighted MRI. (B-J) NanoMnSor ameliorates immunosuppression in TME and exerts synergistic anticancer effects when combined with immunotherapy in orthotopic HCC models. **B** Quantification of mean vessel density in tumors, determined by CD31 and quantitated as percentage of total tumor area at right (*n* = 6–9). CD31-positive ECs were stained red (**C**) Hypoxic tumor areas in orthotopic HCA-1 tumor models after different treatments (*n* = 5–7) are indicated by PIMO-positive staining (green). **D** Treatment with NanoMnSor decreased CD45+ F4/80+ TAMs in tumors (**E**) BMDMs were cultured under normoxic conditions for 24 h with or without NanoMn. Quantitative measures of hydroxyl radicals generated by macrophages after exposure to NanoMn at different doses, (*n* = 3–6). **F** NanoMn increased expression of M1-like genes and decreased M2- like genes in BMDMs (**G**) Treatment of NanoMn and NanoMnSor primed macrophages exhibit M1-like phenotype (*n* = 8–10) and increased cytotoxic CD8+ T cells (**H**) in tumors, as measured by flow cytometry (*n* = 9–17). **I** Increased apoptosis in tumors, indicated by TUNEL staining (green) at 24 days after NanoMnSor treatment. **J** Sizes of orthotopic HCA-1 tumors [156]

Another characteristic feature of cancer cells is the continuous production of lactic acid by aerobic glycolysis which promote the TAM polarization to the M2 phenotype, which increases immunosuppression in the TME and results in hypoxia. Thus, scientists believe that inhibiting the production of lactic acid will efficiently recover the immunosuppressive TME. Recently, the authors designed an RBC-camouflaged hollow MnO_2_ catalytic nanosystem consisting of lactate oxidase and glycolysis inhibitor that consumes lactic acid and generates oxygen concomitantly, which successfully reverses immunosuppression in the TME by significantly reducing the population of M2-type macrophages after combination treatment with PD-L1 [155]. Furthermore, oxygen-producing nanoparticles were also observed to regulate TAM polarization by decreasing hypoxia [192]. Hypoxia promotes not only the invasiveness of tumor cells but also the development of M2-type TAMs, induces a reduction in the number of functional blood vessels, limits the delivery of drugs and is the main cause of drug resistance. To target the vasculature and to revert the hypoxic condition in the TME, Chang et al. designed a tumor-targeted, biodegradable nanodelivery system that contains a MnO_2_ core coated with DOPA, formulated with PLGA to form NanoMn, and loaded with sorafenib (an antiangiogenic drug). They used this strategy to deliver sorafenib and MnO_2_ together to improve the accumulation of sorafenib in hepatocarcinoma and enabled oxygen generation by decreasing hypoxia by the catalytic effect of MnO_2_ on oxygen generation by H_2_O_2_, thus overcoming the resistance to sorafenib. Furthermore, NanoMnSor reprogrammed tumor-promoting macrophages toward immunostimulatory M1 macrophages, increased CD8^+^ cytotoxic T cells in tumors, and amplified the efficacy of the PD-L1 antibody (Fig. 8). This study provides a new strategy of combining antiangiogenic therapies and oxygen generators to modulate the hypoxic TME [156].

**Fig. 9:**
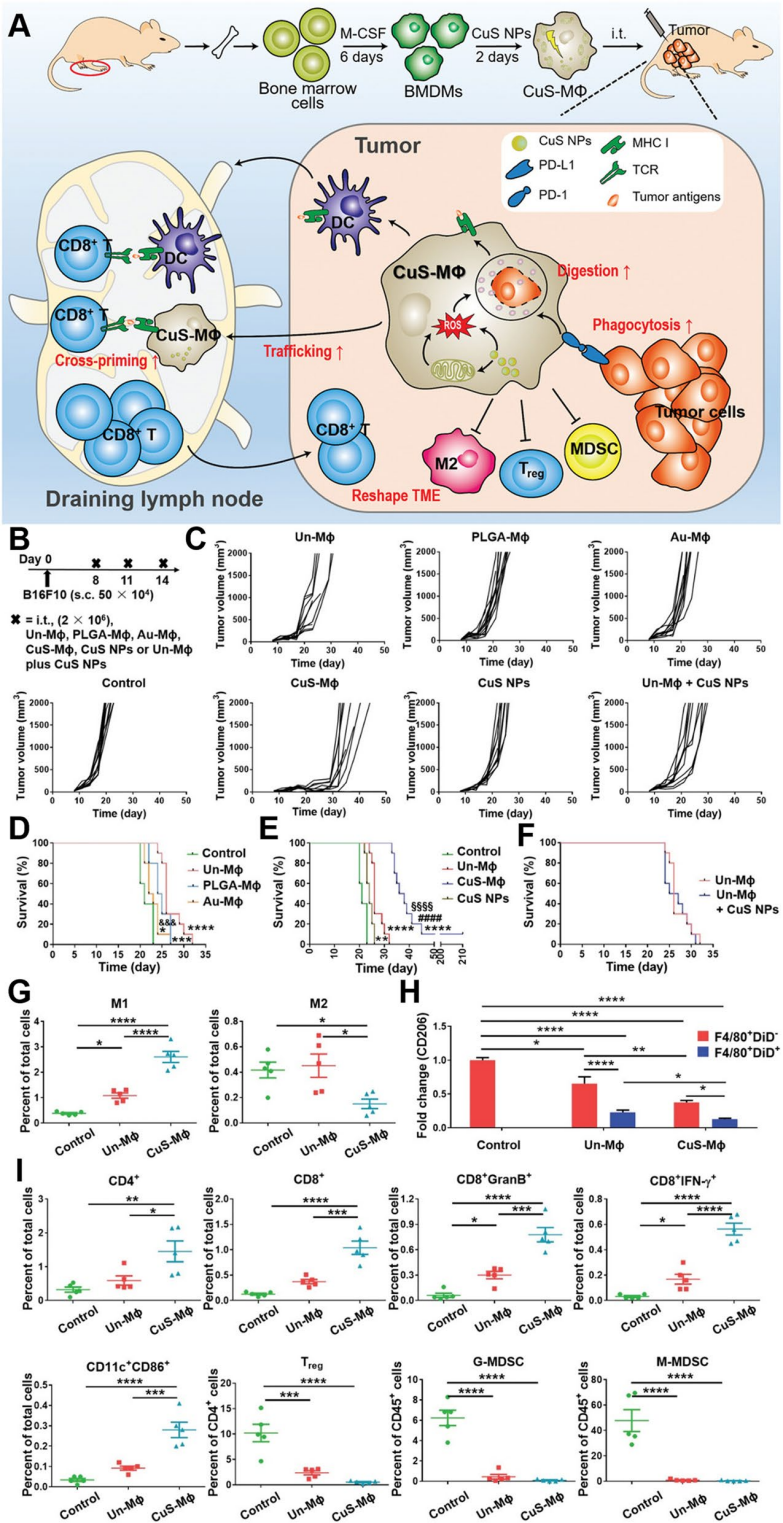
**A** Schematic illustration of redirecting macrophages by CuS NPs for adoptive transfer therapy of solid tumor. **B**-**I** Adoptive transfer of CuS-MΦ for enhanced activity against murine melanoma. **B** Treatment regimen. i.t., Intratumoral injection. **C** Individual B16F10 tumor growth curves following the treatment with Un-MΦ, PLGA-MΦ, Au-MΦ, CuS-MΦ, CuS NPs alone, or Un-MΦ plus CuS NPs (*n* = 10). Control, mice without treatment. For CuS NPs alone group or Un-MΦ plus CuS NPs group, injection dose of CuS NPs was 0.3 μg of Cu, which was equivalent to that of 2 × 106 of CuS-MΦ. **D**-**F** Kaplan-Meier survival curves of selected compared groups, log-rank analysis (*n* = 10). **G** Quantitative analysis of classic macrophages (M1, CD11b + F4/80 + CD206–) versus alternative macrophages (M2, CD11b + F4/80 + CD206+) in tumor on day 20. One-way ANOVA with Tukey’s post-test (*n* = 5). **H** The expression of CD206 in either the transferred (F4/80 + DiD+) or tumor-associated (F4/80 + DiD–) macrophages analyzed on the 3rd day after i.t. transfer of the DiD-labeled CuS-MΦ or Un-MΦ to mice bearing B16F10 tumor, normalized by Control group. One-way ANOVA with Tukey’s post-test (*n* = 5–6). **I** The population of immune cells in the tumor at day 20 including CD4+ T cells, CD8+ T cells, granzyme B-positive CD8+ T cells (CD8 + GranB+), CTLs (CD8 + IFN-γ+), activated DCs (CD11c + CD86+), Treg cells (CD4 + CD25 + Foxp3+), as well as CD11b + Gr-1+ myeloid-derived suppressor cell (MDSC) subsets including CD11b + Gr-1high granulocytic MDSCs (G-MDSCs) and CD11b + Gr-1int monocytic MDSCs (M-MDSCs). One-way ANOVA with Tukey’s post-test (*n* = 5). Data are expressed as mean ± s.e.m. [157]

**Fig. 10:**
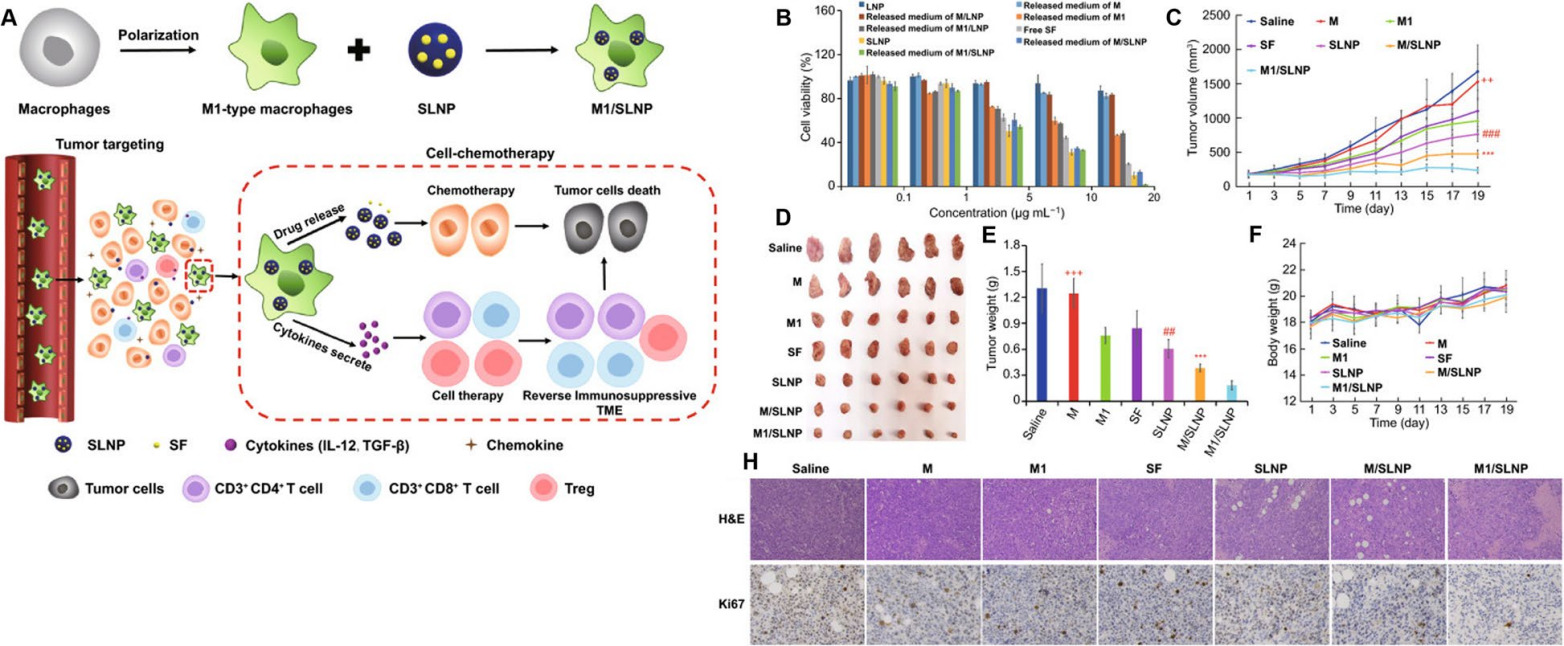
**A** Scheme 1 a Preparation of M1/SLNP. b Schematic illustration of M1/SLNP for tumor targeting delivery to enhance the therapeutic efficiency of HCC, in which dual functional M1-type macrophages as targeting delivery vessel and therapeutic tool. (B-H) M1/SLNP enhanced antitumor efficacy in vitro and in vivo. **B** Cell viability of M1/SLNP in Hepa1–6 cells in vitro. **C** in vivo tumor volume changes. **D** Photographs of tumors. **E** Tumor weights (**F**) Body weight changes from Hepa1–6-bearing mice treated with NS, M, M1, free SF, SLNP, M/SLNP, and M1/SLNP via the tail vein. **H** H&E and Ki67 results of tumor tissues. Magnification: H&E 200×, Ki67 200×. +++p [158].

Adoptive cell therapy (ACT) has been recognized as a promising strategy for cancer treatment [193]. Recently, Xu et al. introduced ACT through copper sulfide nanoparticles (CuS-NPs) that exhibit significant antitumor effects in melanoma-bearing mice. In this study, BMDMs were incubated with PEGylated CuS-NPs and activated by Cu-mediated dynamin-related protein 1 (Drp-1)-mitochondrial fission combined with the Cu-Fenton process, which escalates the production of intracellular reactive oxygen species (ROS) and results in activation of NF-κB, directing BMDM polarization toward the antitumor M1 phenotype (Fig. 9). Furthermore, CuS-NP-stimulated BMDMs demonstrate enhanced phagocytic and digestive capacities by reducing the expression of programmed death-1 (PD-1). Intratumoral transfer of CuS-Mφ reshapes the TME and evokes systemic antitumor immunity, which significantly prolongs the median survival of melanoma-bearing mice [157]. Hou’s group designed a polarized macrophage-based therapy and drug-delivery system for cell chemotherapy by utilizing M1 macrophages, where they act as a therapeutic tool to provide immunotherapy as well as delivery vessels to specifically deliver drugs to tumor cells. M1 macrophages carrying sorafenib (SF) were loaded into lipid nanoparticles (M1/SLNPs). The author demonstrated the increased accumulation of M1/SLNP by tumor sites, which improved the tumor-targeting efficiency of sorafenib and increased the ratio of M1-type macrophages compared to M2 macrophages, ultimately relieving the immunosuppressive TME and inhibiting tumor growth in hepatocellular carcinoma (Fig. 10) [158]. Very recently, scientists developed a nanoparticle-loaded bacterial system, denoted as Ec-PR848, which carries DOX, R848 (TLR7/8 agonist) and bacterial strain MG1655 to target tumor by repolarizing M2 macrophages into M1 macrophages and activate antitumor immunity which ultimately trigger ICD [194]. Researchers have also focused on cytokine-induced killer cell (CIK)-mediated immunotherapy in cancer treatment. In one recent study, the authors combined selenium nanoparticles (SeNPs) with cytokine-induced killer cells (CIK), which effectively increased the infiltration of natural killer cells into tumors and reprogrammed tumor-promoting M2 macrophages to antitumor M1 macrophages, stimulating a strong antitumor response to combat progression. This research proposed a unique method for advancing the therapeutic use of CIK therapy in tumor treatment [159].

STAT3 has been reported to connect oncogenesis and immunological evasion, and its activators (oncostatin M and IL-10) are produced by M2 macrophages in TAMs [195]. Shobaki et al. designed a strategy of targeting TAMs to modulate and modify their functions using lipid nanoparticle formulations (CL4H6-LNPs) loaded with STAT3 siRNA and hypoxia-inducible factor-1α (HIF-1α) siRNA. The siRNA-loaded CL4H6-LNPs exhibited strong blood circulation stability and high tumor-specific accumulation, which induced an antitumor response by silencing two targeted genes, STAT3 and HIF-1α, both of which were found to be increased in TAMs and to promote protumorous functions. The treatment led to increased infiltration of Mφs (CD11b + cells) into the TME and increased levels of M1-type macrophages, which resulted in an outstanding antitumor therapeutic response [160]. Transcription factors such as c-MYC have been found to control the macrophage inflammatory response and their polarization toward the M2 phenotype [196]. Therefore, researchers anticipate that c-MYC inhibitors could inhibit macrophage polarization toward the M2 phenotype. The author developed perfluorocarbon nanoparticles encapsulating a c-MYC inhibitor prodrug (MI3-PD) to specifically target M2 macrophages through integrin αvβ3 with the aim of disrupting M2 polarization without compromising their viability. They showed that these nanoparticles decreased M2 macrophages in the TME without sparing M1-type macrophages [161].

**Fig. 11:**
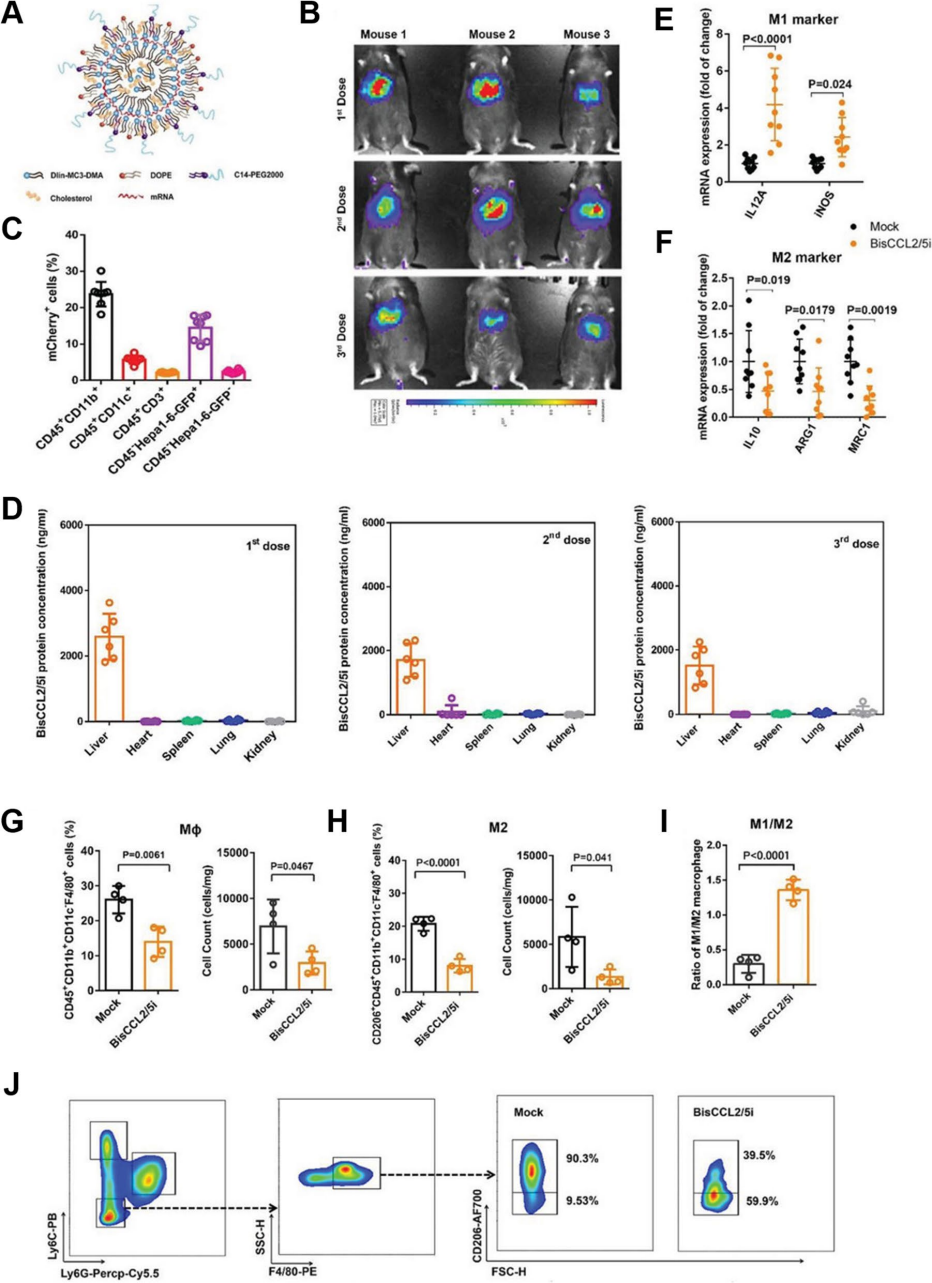
Dual blockade of CCL2 and CCL5 via LNP-mediated mRNA delivery of BisCCL2/5i polarizes macrophage M1 phenotype and reduces the immunosuppression in the TME. **A** Schematic of the mRNA-loaded LNPs. **B** In vivo transfection of Luc mRNA-LNPs after repeated administration (i.v., every 4 days, in total 3 doses). The luciferase was injected into mice 6 h post administration of Luc mRNA-LNPs, followed by measuring luc bioluminescence signal using IVIS imaging, *n* = 3. **C** The quantification of mCherry-positive cells expressed in murine orthotopic HCC tumor tissue 6 h after injection of mCherry mRNA-LNPs (mCherry mRNA: 0.5 mg kg − 1). mRNA is mainly expressed in monocytes (CD45 + CD11b+) and tumor cells (Hepa1–6-GFP+) (*n* = 8). **D** BisCCL2/5i expression in different organs 6 h after each administration of BisCCL2/5i mRNA-LNPs (mRNA: 1 mg kg − 1, i.v., 3 days apart), *n* = 6. The BisCCL2/5i mRNA was mainly expressed in liver tissue and repeated administration resulted in comparable protein level. **E**, **F** mRNA expression of classic M1 (**E**) and M2 (**F**) markers in HCC tumor tissues 48 h after systemic administration of formulated LNPs as a dose corresponding to 1 mg kg − 1 mRNA (Mock, HcRed mRNA). Each data point is an individual sample (*n* = 9); one-way ANOVA and Tukey’s multiple comparisons test. Change of the immunocellular composition in HCC TME 48 h following Mock mRNA-LNPs and BisCCL2/5i mRNA-LNPs treatments (mRNA: 1 mg kg − 1), measured by flow cytometry (*n* = 4; unpaired two-tailed Student’s t-test). **G**, **H** The percentage and cell counts of macrophages (**G**) and their M2 subtype (**H**) in total immune cells. **I**, **J** Representative flow dots of M1- and M2-phenotype macrophages (**I**) and ratio of M1/M2 (**J**). MΦ, macrophages (CD45 + CD11b + CD11c − Ly6C − Ly6G − F4/80+); M2, M2-phenotype macrophages (CD206+). Data are represented as the mean ± s.d. [164]

As described above, CSF-1/CSF1R is known to control macrophage proliferation, differentiation, and migration. However, clinical data have revealed that single-agent treatment targeting the CSF-1R axis has limited efficacy [197]. Recent data also highlight the importance of the mitogen-activated protein kinase (MAPK) pathway in the activation and proliferation of macrophages [198]. To target both CSF-1 and MAPK, Ramesh et al. developed self-assembled dual inhibitor-loaded nanotherapeutics in which a CSF-1R inhibitor and Src homology-region 2 (SHP-2) (to block the “do not eat me” signal) domain phosphatase were coloaded into lipid nanoparticles to simultaneously inhibit the CSF-1R and SHP-2 pathways via the CSF-1 and MAPK pathways. They demonstrated high drug loading, regulated drug release, minimal drug toxicity, superior phagocytic capabilities and hindrance of the CSF-1 and SHP-2 signaling pathways, which stimulates the continuous reprogramming of M2-type macrophages to antitumor M1-type macrophages [162]. Recently, the same author again focused on inhibiting the CSF-1R and MAPK pathways and recently developed a lipid nanoparticle formulation filled with a dual kinase inhibitor, colony-stimulating factor-1 receptor inhibitor and MAPK pathway inhibitor, which inhibited CSF-1R and MAPK signaling to repolarize M2 macrophages to an antitumorigenic M1 phenotype in the TME. They demonstrated suppressed tumor growth and reduced toxicity in a highly aggressive 4 T1 breast cancer model [163]. Given the importance of CCL2 and CCL5 in TAMs [10, 199], recently, researchers bioengineered a single-domain biospecific antibody encapsulated in a clinically approved lipid nanoparticle that binds and neutralizes CCL2-CCL5 by delivering mRNA. The bisCCL2/5i mRNA nanoparticles significantly induced TAM polarization toward the antitumoral M1 type, relieved immunosuppression in TME and, when combined with PD-L1, attained long-term survival in a mouse model of liver, colorectal, and pancreatic cancer (Fig. 11). The mRNA-LNP-based delivery system can be applied to other TAM-enriched cancer types [164]. In recent studies, researchers also focused on CCL19 or macrophage inflammatory protein 3 beta (MIP-3β) to enhance the interaction among immune responses using the targeted gene delivery system with 1,2-dioleoyl-3-trimethylammonium-propane (DOTAP), methoxy poly (ethylene glycol)-poly (lactide) (MPEG-PLA), and folic acid-modified poly (ethylene glycol)-poly(ε-caprolactone) (FA-PEG-PCL) (FDMCA) to polarize macrophages toward M1, inhibiting tumor growth and metastasis in mouse models [165]. The scavenger receptor MARCO (macrophage receptor with collagenous structure) on macrophages has been associated with poor prognosis in breast cancer [200]. Therefore, blocking MARCO with antibodies induces potent antitumor effects by repolarizing TAMs [201] and can be used in combination with nanoparticles.

Recently, Zhang et al. combined ZnO and gold nanoparticles to design a multifunctional nanocomposite, denoted AuNP@mSiO2@DOX-ZnO, that combines the photothermal characteristics of gold nanoparticles, pH-responsive selective drug delivery of ZnO and a chemotherapeutic drug. Their studies revealed that ZnO nanoparticles showed a preference for melanoma cells and caused ICD. Dox- and AuNP@SoO_2_-based photothermal treatment (PTT) showed direct toxicity to cancer cells and contributed to ICD, preventing tumor growth and metastasis [166]. In another study, scientists synthesized AuNPs and CaCO_3_-encapsulated nanoparticles (Au@CaCO_3_) as a stimulant to modulate macrophages. The authors showed the dramatically elevated expression of M2-type macrophages when they were incubated with AuNPs alone. Interestingly, they found that the coincubation of macrophages with Au@CaCO_3_ mediated their reprogramming from tumor-promoting M2 macrophages toward tumoricidal M1-type macrophages, triggering inflammation in macrophages in cancer treatment [167].

**Fig. 12:**
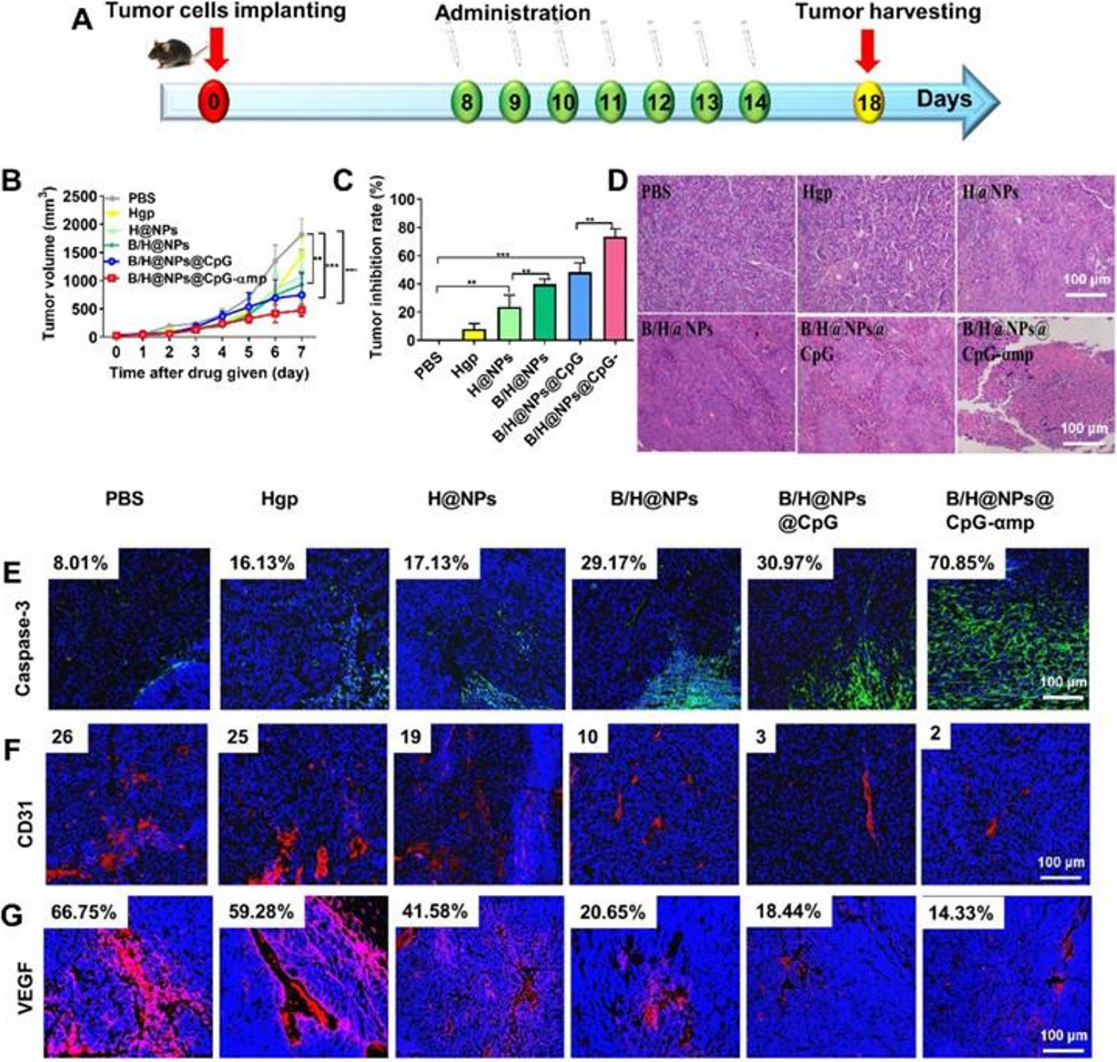
Effective anti-tumor and tumor microenvironment remodeling after treatment with different nano-complexes in the B16 tumor model. **A** Schematic illustration of the time sequence of administration of nano-complex to tumor-bearing mice. **B** Tumor volume from mice that received iv infusion containing different nano-complexes. **C** Tumor inhibition fractions after receiving iv infusion of various nano-complexes formulations. **D** Evidence of necrosis in tumors after treatment with different nano-complexes by hematoxylin and eosin (H&E) staining. **E** Caspase-3 analysis of tumor tissue indicating apoptotic cells by immunofluorescence in frozen tumor sections. **F** The number of vessels per image field is identified by CD31 label after treatment with different nano-complexes. **G** VEGF labeled by immunofluorescence indicates the quality of pro-angiogenesis secretion per image field after treatment with different nano-complexes. The data were analyzed by automatic multispectral imaging system (PerkinElmer Vectra II). Scale bar: 100 μm. Three mice were analyzed in every group (*n* = 3), and one representative image per group is displayed. Data are the mean ± SEM and representative of three independent experiments. Differences between two groups were tested using an unpaired, two-tailed Student’s t-test. Differences among multiple groups were tested with one-way ANOVA followed by Tukey’s multiple comparison. Significant differences between groups are expressed as follows: *P < 0.05, **P < 0.01, or ***P < 0.001 [169]

The active role of hyaluronic acid (HA) in macrophage polarization has already gained attention in cancer immunotherapy. Recently, Rangasami et al. designed supramolecular self-assembled HA-derived immunomodulatory nanoparticles with an anti-inflammatory dexamethasone (DEX) moiety as a hydrophobic moiety to form HA-DEX micelles, which were further loaded with Dox, collectively designated HA-DEX-DOX. HA-DEX-DOX effectively induces the reprogramming of immunosuppressive M2 macrophages toward the M1 phenotype and encourages Dox-mediated apoptosis [168]. In one of the recent studies, researchers developed dual-targeting nanocomplexes, one that targets M2-type macrophages by filling them up with antitumor drugs and another an immunostimulator to remodel the TME. In this nanoformulation, they fabricated PLGA nanoparticles that encapsulate baicalin and tumor-associated antigen, Hgp peptide fragment, while CpG-ODN was adsorbed to a polydopamine coating layer on the surface of nanoparticles. Furthermore, the nanoparticles were coupled with M2-pep and α-pep peptides for dual targeting. The nanoformulation effectively consumes M2-type TAMs and remodels the TME to the proinflammatory type (Fig. 12) [169].

Liver metastasis is associated with activated hepatic cell (aHSC)-mediated fibrosis, and relaxin (RLN), an antifibrotic peptide, acts as a natural regulator to deactivate aHSCs and control liver fibrosis. Recently, amino ethylanisamide (AEAA, potent ligand for sigma-1 receptor, expressed by aHSC tumor)-targeted lipid calcium phosphate (LCP) nanoparticles have been developed to deliver RLN plasmids into aHSC-expressing tumors for the enhanced secretion of RLN protein. They demonstrated that LCP-mediated expression of RLN potently reduced metastasis and shifted the immunosuppressive TME toward the immunostimulatory stage with better cytotoxic T-cell infiltration, which resulted in prolonged animal survival [170]. Recently, Fu et al. developed polymer nanoparticles (P-NPs) by nanoprecipitation methods in which the polymer polystyrene-comaleic anhydride (PSMA) and conjugated polymer poly [2-methoxy-5-(2-ethylhexyloxy)-1,4-phenylenevinylene] (PPV) were coassembled by the nanoprecipitation method, and both acted as critical drivers of macrophage polarization. They reversed the immunosuppressive TME by reprogramming macrophages toward an antitumorous phenotype and inhibiting tumor growth and permitted real-time imaging of nanoparticle uptake by cells and ROS production through PDT to remove residual cells and remaining TAMs [171].

**Fig. 13:**
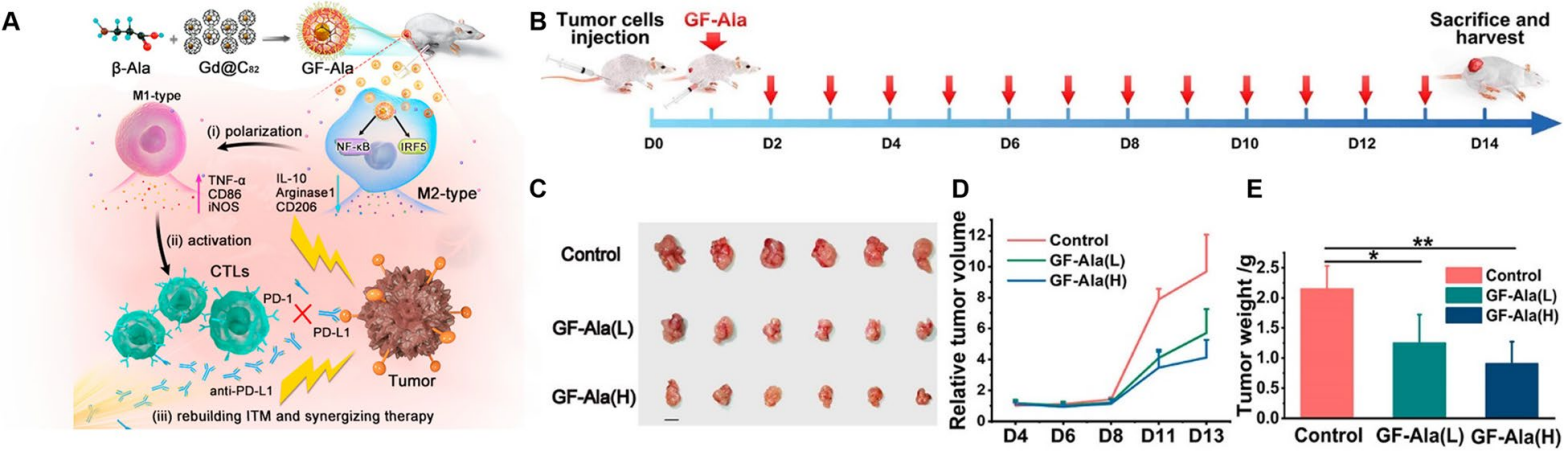
**A** Schematic illustration of the preparation of P-NPs and TAM repolarization effect of P-NPs for immune induced anticancer therapy. **B** Tumor volume in different groups of 4 T1 tumor-bearing mice within 14 days. **p < 0.01. **C** Representative images of tumor tissues were collected from different groups on the 14th day. **D** Body weights of mice after different treatments. **E** Cell viabilities of 4 T1 cancer cells treated with different concentrations of PPV-PSMA-NPs with or without irradiation (30 min, 25 mM cm^− 2^). **F** Representative immunofluorescence staining images for CD80 (red), iNOS (red), TNFα (red), CD206 (green) and CD163 (green), as well as immunofluorescence staining observation of TUNEL staining (green) of tumor sections from different groups [172]

Rebuilding an immunosuppressive TME by reprogramming TAMs to a tumoricidal state and boosting the body’s natural immune system has already gained much attention. One recent study revealed that Gd@C82 nanoparticles modified with β-alanines (GF-Ala) remodel the immunosuppressive TME by reprogramming the tumor-supportive M2 phenotype to antitumor M1 macrophages by activating the NF-κB and IRF5 pathways (Fig. 13), which trigger an antitumor response by inducing the infiltration of cytotoxic T-lymphocytes and inhibiting tumor growth [172].

**Fig. 14:**
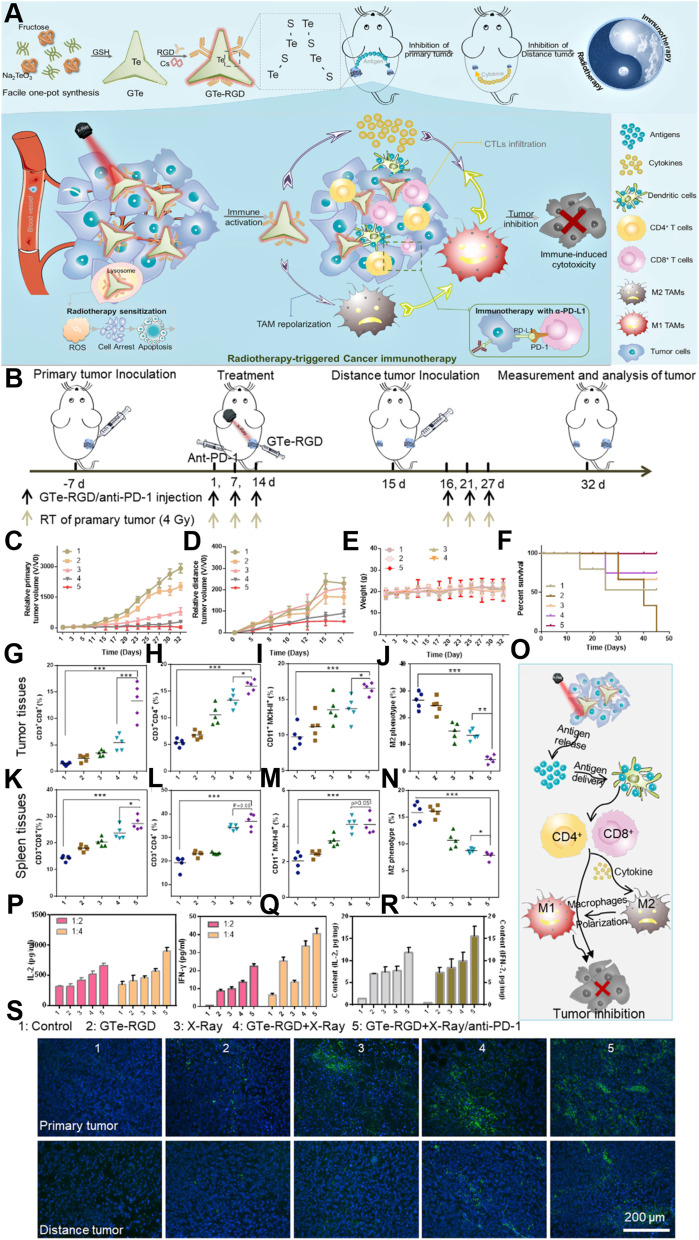
**A** Schematic diagram shows the facile synthesis of triangle-shaped Te nanostar (GTe-RGD) and its combination with checkpoint blocking as an excellent radio sensitizer for boosting immunotherapy, which may provide reasonable evidence of the synergistic effect of RT and immunotherapy. (B-S) In Vivo GTe-RGD-Enhanced RT for Boosting Checkpoint Blockade Immunotherapy. **B** Schematic diagram of our experiments and the overall survival curves of mice with different treatments (experimental design to evaluate the enhanced cancer RT combined with anti-PD-1 using a bilateral subcutaneous 4 T1 tumor model). Tumors on the right legs were referred to as “primary tumors” and received X-ray treatment, while left tumors were called “distant tumors” and did not undergo RT. Average growth of (**C**) primary tumors and (**D**) distant tumors in mice receiving various treatments. **E** and **F** Body weight and overall Kaplan-Meier survival curves of 4 T1 tumor-bearing mice in different groups after various treatments. **G** and **H–N** Flow-cytometry analysis of immune cells including CD8^+^ T cells, CD4+ T cells, DCs, and M2 phenotype macrophages in tumor and spleen tissues (*n* = 5 per group). **S** Immunofluorescence analysis of CD8 antibody (green, CD8+ T cells) and DAPI (blue, cell nuclei) in primary and distant tumor tissues. (O) Mechanism of anticancer immune responses induced by GTe-RGD-based RT in combination with checkpoint blockade. **P** and **Q** Levels of IL-2 and IFN-g secreted by T lymphocytes stimulated with different proportions of dead cancer cells in different treatment groups. **R** Serum cytokine concentrations in mice after different treatments. All data are presented as mean G SD (*n* = 5). *p < 0.05, **p < 0.01, ***p < 0.001 [174]

Due to the enhanced permeability and retention (EPR) effect, nanoparticle-based treatment confers prolonged blood circulation time, improves tumor targeting efficacy, and reduces off-target effects. Recently, researchers employed hydralazine (HDZ), an antihypertension vasodilator, which is thought to dilate vessels and promote the penetration of nanoparticles more deeply in advanced tumors. They prepared a DiD-loaded liposomal formulation of HDZ, which significantly increased nanoparticle aggregation and penetration in tumors and repolarized TAMs by normalizing tumor blood vessels, which effectively inhibited the growth of desmoplastic melanoma [173]. Presently, researchers synthesize tellurium nanosine (GTE-RGD) by a one-pot hydrothermal method and combine it with radiotherapy and checkpoint blockade in a breast cancer mouse model. GTE-RGD potentiates radiotherapy, which leads to tumor eradication and enhances cytotoxic T lymphocytes, elicits antitumor immunity and inhibits metastasis (Fig. 14). Additionally, nanoparticles were able to efficiently reduce the population of M2-type TAMs and provided an attractive clinical alternative for tumor treatment [174].

**Fig. 15:**
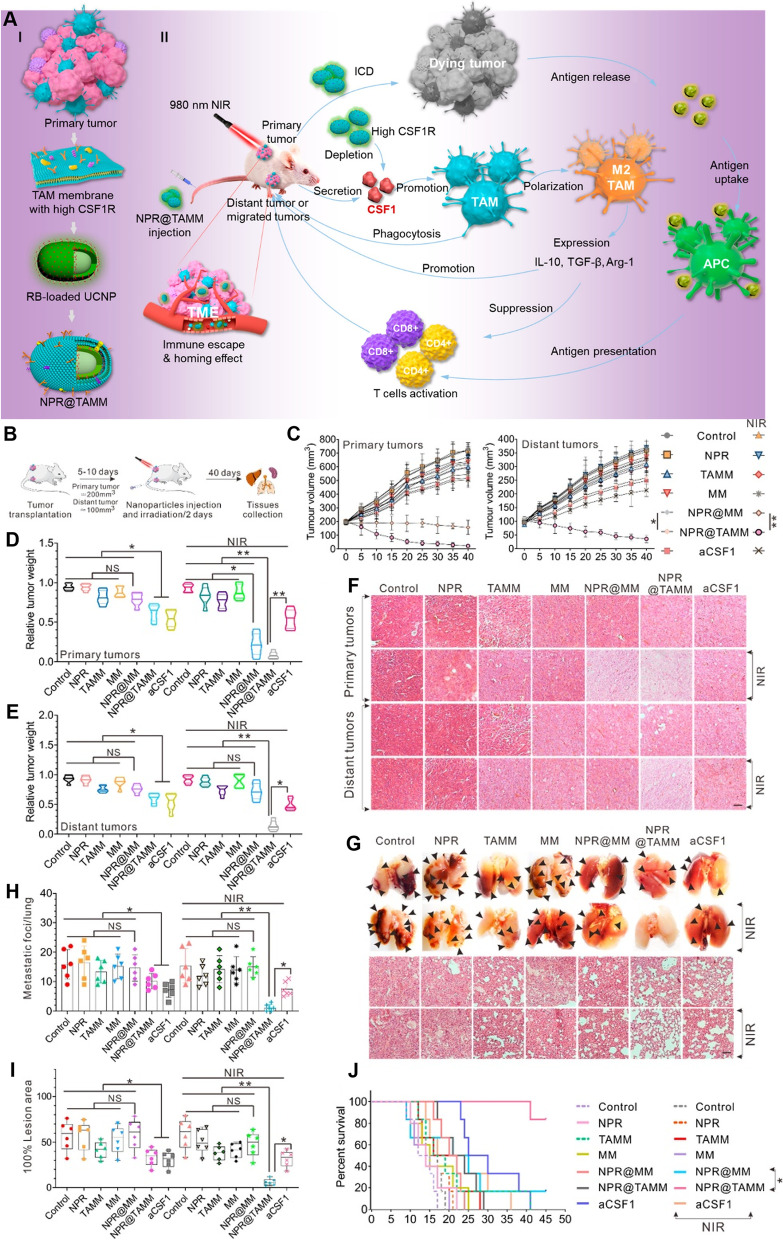
**A** Schematic illustration of the tumor-associated-macrophage-membrane-coated up conversion nanoparticles for improved photodynamic immunotherapy. **B-J** In vivo antitumor therapeutic effects. **B** Schematic illustration of 4 T1 tumor model establishment and the therapeutic regimen. **C** Tumor growth curves for primary tumor and distant tumor. **D** Tumor weight for primary tumor. **E** Tumor weight for distant tumor. **F** Histological analysis of H&E staining for primary and distant tumor. **G** Photographs show representative external views of lung with the histological analysis of H&E staining. Arrows indicate focal tumor nodules on lung surfaces. Scale bar = 100 μm. (H, I) Graphs show the quantification of metastatic foci (**H**) and lesion area (**I**) in the different treatment groups from part f. **J** The survival curve of tumor-bearing mice calculated by Kaplan−Meier estimate. Data are means ± SD. *P < 0.05; **P < 0.01. NS, no significance. *n* = 6/group [203]

Currently, cell membrane-coated nanoparticles, such as red blood cells or natural killer cell membrane-coated nanoparticles, have been steadily utilized in cancer immunotherapy, achieving satisfactory preventive and therapeutic efficacy due to autologous multiantigen presentation and homotypic targeting [175]. Recently, researchers designed the macrophage membrane-coated, TMP195 (TAM repolarization agent)-containing polydopamine nanoparticles to target the inflammatory environment in post-PTT residual tumor tissue, which relieved the immunosuppressive TME and allowed for complete tumor eradication by rescuing T cells [202]. Another recent study by Chen et al. reported a tumor-derived TAM membrane (TAMM) with antigen-homing ability and compatibility to block CSF-1 produced by tumor cells in the TME and inhibit the interactions between TAMs and tumors. Briefly, TAM-like upconversion nanophotosensitizers, denoted NPR@TAMM, have been developed as a potential cancer photodynamic therapy and shifted macrophages from an M2-like phenotype to an inflammatory M1-like state, inducing ICD (Fig. 15), consequently enhancing the antitumor immune response by activating antigen-presenting cells to enhance the production of tumor-specific effector T cells in tumors and offering new opportunities to explore endogenous TAMs as delivery vehicles with potential as personalized tumor therapies [203].

## Conclusions

TAMs are TME-resident innate immune cells that contribute to tumor development and progression. Therapeutic agents that deplete TAMs, block their recruitment or activate their repolarization toward the antitumoral M1 type have shown great potential for clinical applications [48]. However, loss of the immunostimulatory role of macrophages as phagocytes and professional antigen-presenting cells in the TME is an unavoidable disadvantage of the first two methods and may result in unpredictable and multifaceted stromal reactions in hosts. In contrast, with reprogramming, TAMs can be transformed into proinflammatory macrophages to attenuate their immunosuppressive ability while enhancing their immunostimulatory functions. Recent advancements in nanobiotechnology have allowed the incorporation of several diagnostic and therapeutic agents into nanoparticles. As professional antigen-presenting cells, macrophages bridge the gap between innate and adaptive immunity. The abundantly expressed surface receptors on TAMs, such as mannose, folate, SIGLEC1, or scavenger receptors, could represent novel therapeutic targets [95, 136, 137, 139, 146]. In solid tumors, tumor cells are deeply resident, which usually diminishes the therapeutic effects of drugs. Nanomedicine improves the stability and localization of anticancer drugs and decreases the toxicity of drugs in healthy tissues. After eating up the nanoparticles, TAMs distribute them to the entire tumor, including the hypoxic region, which enhances the distribution and permeability of nanoparticles or drug-loaded nanoparticles in the tissues [156]. Moreover, TAMs can also be employed as carriers for nanoparticles in live-cell therapies to enhance their tumor-killing ability [153]. A number of TME-sensitive targeted preparations may also be designed using a range of physiological differences between the TME and normal tissue, such as low pH, hypoxia, or increased expression of specific enzymes such as MMPs [136, 138, 150].

While nanoparticle-targeting macrophages have resulted in some successes in cancer immunotherapy and drug delivery, there are still various intriguing issues and obstacles to overcome. Due to the extraordinary heterogeneity of TAMs, the time course of macrophage recruitment and polarization status is not clear. Macrophage infiltration is also dependent on the disease severity and stage. The use of macrophage-targeting probes in molecular imaging to determine the status of macrophages in the TME seems promising [204]. The similarity in receptors on normal cells can cause failure in targeting specific targets on immunosuppressive macrophages, resulting in side effects and toxicity. Another critical issue is that nanomedicine-based strategies usually rely on the upregulated expression of TAM receptors, which typically varies with tumor progression. Interactions between cells and nanoparticles, such as internalization and processing of nanoparticles by macrophages and mechanistic studies on the effects of nanoparticles on macrophages, are also essential mechanisms that must be clarified. The immune milieu in the tumor is complex, and TAM-based cancer immunotherapies alone are insufficient to eliminate tumors. Combining these with another immunotherapy, such as PD-L1 or CAR-based treatment, or chemotherapy and radiation is an effective way to tackle this problem. To produce synergistic activity and avoid significant adverse effects, combination therapy must be carefully designed based on the characteristics of nanoparticles and the TME. If we address these obstacles and design nanoparticles with better targeting, macrophages will be a potent weapon that can overcome the problems related to solid tumors, and they will be promising for clinical applications.

## Data Availability

All data generated in this review are available from the corresponding author upon reasonable request.

## Abbreviations

TAMs: Tumor-associated macrophages
TME: Tumor microenvironment
BMDM: Bone-marrow derived macrophages
TRM: Tissue-resident macrophages
CCL2: C-C motif chemokine ligand 2
CSF-1: Colony-stimulating factor-1
GM-CSF: Granulocyte-macrophage colony-stimulating factor
TNF-α: Tumor necrosis factor-alpha
IL: Interleukins
MHC: Major histocompatibility complex
EGFR: Epidermal growth factor receptor
EMT: Endothelial-mesenchymal transition
VEGF: Vascular endothelial growth factor
PDGF: Platelet-derived growth factor
TGF-ß: Transforming growth factor-ß
FGF: Fibroblast growth factor
ECM: Extracellular matrix
STAT3: Signal transducer and activator of transcription 3
TRAIL: Tumor necrosis factor-related apoptosis-inducing ligand
PI3K: Phosphoinositide 3 kinase
PGE2: Prostaglandin E2
Arg-1: Arginase-1
PD-L1: Programmed death-ligand 1
TREM2: Triggering receptor expressed on myeloid cells 2
JAK: Janus tyrosine kinase
GSTP1: Glutathione S transferase P1
NSCLC: Non-small lung cancer
MIP-3ß: Macrophage inflammatory protein-3ß
PDAC: Pancreatic ductal adenocarcinoma
iPSC: Induced pluripotent stem cell
CAR: Chimeric antigen receptor
CAR-Ps: CAR for phagocytosis
CAR-M: CAR macrophages
CDNP: ß-cyclodextrin nanoparticles
IMDQ: Imidazoquinolinone
SIRP: Signal regulatory protein
HA: Hyaluronic acid
ZnO: Zinc oxide
AuNPs: Gold nanoparticles
IONP: Iron-oxide nanoparticles
DEX: Dexamethasone
DOX: Doxorubicin
ACT: Adoptive cell transfer
CIK: Cytokine-induced killer cells

## References

[1] Kennedy LB, Salama AKS (2020). A review of cancer immunotherapy toxicity. CA Cancer J Clin.

[2] Vanneman M, Dranoff G (2012). Combining immunotherapy and targeted therapies in cancer treatment. Nat Rev Cancer.

[3] Nam J, Son S, Park KS, Zou WP, Shea LD, Moon JJ (2019). Cancer nanomedicine for combination cancer immunotherapy. Nat Rev Mater.

[4] Cassetta L, Pollard JW (2018). Targeting macrophages: therapeutic approaches in cancer. Nat Rev Drug Discov.

[5] DeNardo DG, Ruffell B (2019). Macrophages as regulators of tumour immunity and immunotherapy. Nat Rev Immunol.

[6] DiPietro LA, Wilgus TA, Koh TJ (2021). Macrophages in healing wounds: paradoxes and paradigms. Int J Mol Sci.

[7] Locati M, Mantovani A, Sica A (2013). Macrophage activation and polarization as an adaptive component of innate immunity. Dev Funct Myeloid Subsets.

[8] Mantovani A, Ponzetta A, Inforzato A, Jaillon S (2019). Innate immunity, inflammation and tumour progression: double-edged swords. J Intern Med.

[9] Murray PJ, Wynn TA (2011). Protective and pathogenic functions of macrophage subsets. Nat Rev Immunol.

[10] Mantovani A, Marchesi F, Malesci A, Laghi L, Allavena P (2017). Tumour-associated macrophages as treatment targets in oncology. Nat Rev Clin Oncol.

[11] Ruffell B, Coussens LM (2015). Macrophages and therapeutic resistance in Cancer. Cancer Cell.

[12] Kitano Y, Okabe H, Yamashita Y, Nakagawa S, Saito Y, Umezaki N, Tsukamoto M, Yamao T, Yamamura K, Arima K (2018). Tumour-infiltrating inflammatory and immune cells in patients with extrahepatic cholangiocarcinoma. Br J Cancer.

[13] Gubin MM, Esaulova E, Ward JP, Malkova ON, Runci D, Wong P, Noguchi T, Arthur CD, Meng W, Alspach E (2018). High-dimensional analysis delineates myeloid and lymphoid compartment remodeling during successful immune-checkpoint Cancer therapy. Cell.

[14] Molgora M, Esaulova E, Vermi W, Hou JC, Chen Y, Luo JQ, Brioschi S, Bugatti M, Omodei AS, Ricci B (2020). TREM2 modulation remodels the tumor myeloid landscape enhancing anti-PD-1 immunotherapy. Cell.

[15] Xiong HZ, Mittman S, Rodriguez R, Moskalenko M, Pacheco-Sanchez P, Yang YG, Nickles D, Cubas R (2019). Anti-PD-L1 treatment results in functional remodeling of the macrophage compartment. Cancer Res.

[16] Ferrari M (2005). Cancer nanotechnology: opportunities and challenges. Nat Rev Cancer.

[17] Biju V, Itoh T, Anas A, Sujith A, Ishikawa M (2008). Semiconductor quantum dots and metal nanoparticles: syntheses, optical properties, and biological applications. Anal Bioanal Chem.

[18] Mitchell MJ, Billingsley MM, Haley RM, Wechsler ME, Peppas NA, Langer R (2021). Engineering precision nanoparticles for drug delivery. Nat Rev Drug Discov.

[19] Aryal S, Key J, Stigliano C, Landis MD, Lee DY, Decuzzi P (2014). Positron emitting magnetic Nanoconstructs for PET/MR imaging. Small.

[20] Israel LL, Galstyan A, Holler E, Ljubimova JY (2020). Magnetic iron oxide nanoparticles for imaging, targeting and treatment of primary and metastatic tumors of the brain. J Control Release.

[21] Woodman C, Vundu G, George A, Wilson CM (2021). Applications and strategies in nanodiagnosis and nanotherapy in lung cancer. Semin Cancer Biol.

[22] Raju GSR, Benton L, Pavitraa E, Yu JS (2015). Multifunctional nanoparticles: recent progress in cancer therapeutics. Chem Commun.

[23] Shi J, Kantoff PW, Wooster R, Farokhzad OC (2017). Cancer nanomedicine: progress, challenges and opportunities. Nat Rev Cancer.

[24] Zhao ZW, Zheng LY, Chen WQ, Weng W, Song JJ, Ji JS (2019). Delivery strategies of cancer immunotherapy: recent advances and future perspectives. J Hematol Oncol.

[25] Sylvestre M, Crane CA, Pun SH (2020). Progress on modulating tumor-associated macrophages with biomaterials. Adv Mater.

[26] Ovais M, Guo MY, Chen CY (2019). Tailoring nanomaterials for targeting tumor-associated macrophages. Adv Mater.

[27] Zanganeh S, Hutter G, Spitler R, Lenkov O, Mahmoudi M, Shaw A, Pajarinen JS, Nejadnik H, Goodman S, Moseley M (2016). Iron oxide nanoparticles inhibit tumour growth by inducing pro-inflammatory macrophage polarization in tumour tissues. Nat Nanotechnol.

[28] Zhang Y, Chen YL, Li JH, Zhu XQ, Liu YJ, Wang XX, Wang HF, Yao YJ, Gao YF, Chen ZZ (2021). Development of toll-like receptor agonist-loaded nanoparticles as precision immunotherapy for reprogramming tumor-associated macrophages. ACS Appl Mater Interfaces.

[29] Miller MA, Zheng YR, Suresh GW, Pfirschke C, Zope H, Engblom C, Kohler RH, Iwamoto Y, Yang KS, Askevold B (2015). Tumour-associated macrophages act as a slow-release reservoir of nano-therapeutic Pt(IV) pro-drug. Nat Commun.

[30] Wynn TA, Chawla A, Pollard JW (2013). Macrophage biology in development, homeostasis and disease. Nature.

[31] Satpathy AT, Wu XD, Albring JC, Murphy KM (2012). Re(de)fining the dendritic cell lineage. Nat Immunol.

[32] Cortez-Retamozo V, Etzrodt M, Newton A, Rauch PJ, Chudnovskiy A, Berger C, Ryan RJH, Iwamoto Y, Marinelli B, Gorbatov R (2012). Origins of tumor-associated macrophages and neutrophils. Proc Natl Acad Sci U S A.

[33] Ginhoux F, Guilliams M (2016). Tissue-resident macrophage ontogeny and homeostasis. Immunity.

[34] Guerriero JL (2018). Macrophages: the road less traveled, changing anticancer therapy. Trends Mol Med.

[35] Mezu-Ndubuisi OJ, Maheshwari A (2021). Role of macrophages in fetal development and perinatal disorders. Pediatr Res.

[36] Hoeffel G, Chen JM, Lavin Y, Low D, Almeida FF, See P, Beaudin AE, Lum J, Low I, Forsberg EC (2015). C-Myb(+) Erythro-myeloid progenitor-derived fetal monocytes give rise to adult tissue-resident macrophages. Immunity.

[37] Ginhoux F, Greter M, Leboeuf M, Nandi S, See P, Gokhan S, Mehler MF, Conway SJ, Ng LG, Stanley ER (2010). Fate mapping analysis reveals that adult microglia derive from primitive macrophages. Science.

[38] Gibbings SL, Goyal R, Desch AN, Leach SM, Prabagar M, Atif SM, Bratton DL, Janssen W, Jakubzick CV (2015). Transcriptome analysis highlights the conserved difference between embryonic and postnatal-derived alveolar macrophages. Blood.

[39] Loyher PL, Hamon P, Laviron M, Meghraoui-Kheddar A, Goncalves E, Deng ZH, Torstensson S, Bercovici N, de Chanville CB, Combadiere B (2018). Macrophages of distinct origins contribute to tumor development in the lung. J Exp Med.

[40] Zhu Y, Herndon JM, Sojka DK, Kim KW, Knolhoff BL, Zuo C, Cullinan DR, Luo JQ, Bearden AR, Lavine KJ (2017). Tissue-resident macrophages in pancreatic ductal adenocarcinoma originate from embryonic hematopoiesis and promote tumor progression. Immunity.

[41] Strachan DC, Ruffell B, Oei Y, Bissell MJ, Coussens LM, Pryer N, Daniel D (2013). CSF1R inhibition delays cervical and mammary tumor growth in murine models by attenuating the turnover of tumor-associated macrophages and enhancing infiltration by CD8(+) T cells. Oncoimmunology.

[42] Casanova-Acebes M, Dalla E, Leader AM, LeBerichel J, Nikolic J, Morales BM, Brown M, Chang C, Troncoso L, Chen ST (2021). Tissue-resident macrophages provide a pro-tumorigenic niche to early NSCLC cells. Nature.

[43] Etzerodt A, Moulin M, Doktor TK, Delfini M, Mossadegh-Keller N, Bajenoff M, Sieweke MH, Moestrup SK, Auphan-Anezin N, Lawrence T (2020). Tissue-resident macrophages in omentum promote metastatic spread of ovarian cancer. J Exp Med.

[44] Krishnan V, Schaar B, Tallapragada S, Dorigo O (2018). Tumor associated macrophages in gynecologic cancers. Gynecol Oncol.

[45] Sica A, Mantovani A (2012). Macrophage plasticity and polarization: in vivo veritas. J Clin Invest.

[46] Mantovani A, Sozzani S, Locati M, Allavena P, Sica A (2002). Macrophage polarization: tumor-associated macrophages as a paradigm for polarized M2 mononuclear phagocytes. Trends Immunol.

[47] Vitale I, Manic G, Coussens LM, Kroemer G, Galluzzi L (2019). Macrophages and metabolism in the tumor microenvironment. Cell Metab.

[48] Pathria P, Louis TL, Varner JA (2019). Targeting tumor-associated macrophages in Cancer. Trends Immunol.

[49] Yuan X, Zhang J, Li D, Mao Y, Mo F, Du W, Ma X (2017). Prognostic significance of tumor-associated macrophages in ovarian cancer: a meta-analysis. Gynecol Oncol.

[50] Zhao X, Qu J, Sun Y, Wang J, Liu X, Wang F, Zhang H, Wang W, Ma X, Gao X, Zhang S (2017). Prognostic significance of tumor-associated macrophages in breast cancer: a meta-analysis of the literature. Oncotarget.

[51] Yang Z, Zhang M, Peng R, Liu J, Wang F, Li Y, Zhao Q, Liu J (2020). The prognostic and clinicopathological value of tumor-associated macrophages in patients with colorectal cancer: a systematic review and meta-analysis. Int J Color Dis.

[52] Komohara Y, Niino D, Ohnishi K, Ohshima K, Takeya M (2015). Role of tumor-associated macrophages in hematological malignancies. Pathol Int.

[53] Komohara Y, Jinushi M, Takeya M (2014). Clinical significance of macrophage heterogeneity in human malignant tumors. Cancer Sci.

[54] Wang J, Li DY, Cang HX, Guo B (2019). Crosstalk between cancer and immune cells: role of tumor-associated macrophages in the tumor microenvironment. Cancer Med.

[55] Cassetta L, Fragkogianni S, Sims AH, Swierczak A, Forrester LM, Zhang H, Soong DYH, Cotechini T, Anur P, Lin EY (2019). Human tumor-associated macrophage and monocyte transcriptional landscapes reveal Cancer-specific reprogramming, biomarkers, and therapeutic targets. Cancer Cell.

[56] Helm O, Held-Feindt J, Grage-Griebenow E, Reiling N, Ungefroren H, Vogel I, Kruger U, Becker T, Ebsen M, Rocken C (2014). Tumor-associated macrophages exhibit pro- and anti-inflammatory properties by which they impact on pancreatic tumorigenesis. Int J Cancer.

[57] Debebe A, Medina V, Chen CY, Mahajan IM, Jia C, Fu D, He L, Zeng N, Stiles BW, Chen CL (2017). Wnt/beta-catenin activation and macrophage induction during liver cancer development following steatosis. Oncogene.

[58] Li XL, Liu R, Su X, Pan YS, Han XF, Shao CS, Shi YF (2019). Harnessing tumor-associated macrophages as aids for cancer immunotherapy. Mol Cancer.

[59] Llovet JM, Zucman-Rossi J, Pikarsky E, Sangro B, Schwartz M, Sherman M, Gores G (2016). Hepatocellular carcinoma. Nat Rev Dis Primers.

[60] Xia LL, Zhu XH, Zhang L, Xu YH, Chen GP, Luo J (2020). EZH2 enhances expression of CCL5 to promote recruitment of macrophages and invasion in lung cancer. Biotechnol Appl Biochem.

[61] Lugano R, Ramachandran M, Dimberg A (2020). Tumor angiogenesis: causes, consequences, challenges and opportunities. Cell Mol Life Sci.

[62] Bosurgi L, Cao YG, Cabeza-Cabrerizo M, Tucci A, Hughes LD, Kong Y, Weinstein JS, Licona-Limon P, Schmid ET, Pelorosso F (2017). Macrophage function in tissue repair and remodeling requires IL-4 or IL-13 with apoptotic cells. Science.

[63] Viallard C, Larrivee B (2017). Tumor angiogenesis and vascular normalization: alternative therapeutic targets. Angiogenesis.

[64] Gocheva V, Wang HW, Gadea BB, Shree T, Hunter KE, Garfall AL, Berman T, Joyce JA (2010). IL-4 induces cathepsin protease activity in tumor-associated macrophages to promote cancer growth and invasion. Genes Dev.

[65] Wang W, Liu Y, Guo J, He H, Mi X, Chen C, Xie J, Wang S, Wu P, Cao F (2018). miR-100 maintains phenotype of tumor-associated macrophages by targeting mTOR to promote tumor metastasis via Stat5a/IL-1ra pathway in mouse breast cancer. Oncogenesis.

[66] Huang R, Wang S, Wang N, Zheng Y, Zhou J, Yang B, Wang X, Zhang J, Guo L, Wang S (2020). CCL5 derived from tumor-associated macrophages promotes prostate cancer stem cells and metastasis via activating beta-catenin/STAT3 signaling. Cell Death Dis.

[67] Liu W, Wang WJ, Wang XR, Xu C, Zhang N, Di W (2020). Cisplatin-stimulated macrophages promote ovarian cancer migration via the CCL20-CCR6 axis. Cancer Lett.

[68] Liu Q, Yang C, Wang S, Shi D, Wei C, Song J, Lin X, Dou R, Bai J, Xiang Z (2020). Wnt5a-induced M2 polarization of tumor-associated macrophages via IL-10 promotes colorectal cancer progression. Cell Commun Signal.

[69] Kitamura T, Qian BZ, Soong D, Cassetta L, Noy R, Sugano G, Kato Y, Li JF, Pollard JW (2015). CCL2-induced chemokine cascade promotes breast cancer metastasis by enhancing retention of metastasis-associated macrophages. J Exp Med.

[70] Qian BZ, Zhang H, Li JF, He TF, Yeo EJ, Soong DYH, Carragher NO, Munro A, Chang A, Bresnick AR (2015). FLT1 signaling in metastasis-associated macrophages activates an inflammatory signature that promotes breast cancer metastasis. J Exp Med.

[71] Wu JD, Gao W, Tang QY, Yu Y, You W, Wu ZS, Fan Y, Zhang L, Wu C, Han GY (2021). M2 macrophage-derived exosomes facilitate HCC metastasis by transferring alpha(M)beta(2) integrin to tumor cells. Hepatology.

[72] Chen Q, Zhang XHF, Massague J (2011). Macrophage binding to receptor VCAM-1 transmits survival signals in breast Cancer cells that invade the lungs. Cancer Cell.

[73] Banerjee P, Zhang R, Ivan C, Galletti G, Clise-Dwyer K, Barbaglio F, Scarfo L, Aracil M, Klein C, Wierda W (2019). Trabectedin reveals a strategy of immunomodulation in chronic lymphocytic leukemia. Cancer Immunol Res.

[74] Yin Z, Ma TT, Huang BW, Lin LH, Zhou Y, Yan JH, Zou YP, Chen S (2019). Macrophage-derived exosomal microRNA-501-3p promotes progression of pancreatic ductal adenocarcinoma through the TGFBR3-mediated TGF-beta signaling pathway. J Exp Clin Cancer Res.

[75] Klimp AH, Hollema H, Kempinga C, van der Zee AGJ, de Vries EGE, Daemen T (2001). Expression of cyclooxygenase-2 and inducible nitric oxide synthase in human ovarian tumors and tumor-associated macrophages. Cancer Res.

[76] Bronte V, Brandau S, Chen SH, Colombo MP, Frey AB, Greten TF, Mandruzzato S, Murray PJ, Ochoa A, Ostrand-Rosenberg S (2016). Recommendations for myeloid-derived suppressor cell nomenclature and characterization standards. Nat Commun.

[77] Veglia F, Sanseviero E, Gabrilovich DI (2021). Myeloid-derived suppressor cells in the era of increasing myeloid cell diversity. Nat Rev Immunol.

[78] Beury DW, Parker KH, Nyandjo M, Sinha P, Carter KA, Ostrand-Rosenberg S (2014). Cross-talk among myeloid-derived suppressor cells, macrophages, and tumor cells impacts the inflammatory milieu of solid tumors. J Leukoc Biol.

[79] Kwak T, Wang F, Deng H, Condamine T, Kumar V, Perego M, Kossenkov A, Montaner LJ, Xu XW, Xu W (2020). Distinct populations of immune-suppressive macrophages differentiate from Monocytic myeloid-derived suppressor cells in Cancer. Cell Rep.

[80] Kumar V, Cheng PY, Condamine T, Mony S, Languino LR, McCaffrey JC, Hockstein N, Guarino M, Masters G, Penman E (2016). CD45 phosphatase inhibits STAT3 transcription factor activity in myeloid cells and promotes tumor-associated macrophage differentiation. Immunity.

[81] Mantovani A, Barajon I, Garlanda C (2018). IL-1 and IL-1 regulatory pathways in cancer progression and therapy. Immunol Rev.

[82] Majety M, Runza V, Lehmann C, Hoves S, Ries CH (2018). A drug development perspective on targeting tumor-associated myeloid cells. FEBS J.

[83] La Fleur L, Boura VF, Alexeyenko A, Berglund A, Ponten V, Mattsson JSM, Djureinovic D, Persson J, Brunnstrom H, Isaksson J (2018). Expression of scavenger receptor MARCO defines a targetable tumor-associated macrophage subset in non-small cell lung cancer. Int J Cancer.

[84] Barclay AN, van den Berg TK (2014). The interaction between signal regulatory protein alpha (SIRP alpha) and CD47: structure, function, and therapeutic target. Annu Rev Immunol.

[85] Guerriero JL, Sotayo A, Ponichtera HE, Castrillon JA, Pourzia AL, Schad S, Johnson SF, Carrasco RD, Lazo S, Bronson RT (2017). Class IIa HDAC inhibition reduces breast tumours and metastases through anti-tumour macrophages. Nature.

[86] Vergadi E, Ieronymaki E, Lyroni K, Vaporidi K, Tsatsanis C (2017). Akt signaling pathway in macrophage activation and M1/M2 polarization. J Immunol.

[87] D'Errico G, Alonso-Nocelo M, Vallespinos M, Hermann PC, Alcala S, Garcia CP, Martin-Hijano L, Valle S, Earl J, Cassiano C (2019). Tumor-associated macrophage-secreted 14-3-3 zeta signals via AXL to promote pancreatic cancer chemoresistance. Oncogene.

[88] Gyori D, Lim EL, Grant FM, Spensberger D, Roychoudhuri R, Shuttleworth SJ, Okkenhaug K, Stephens LR, Hawkins PT (2018). Compensation between CSF1R(+) macrophages and Foxp3(+) Treg cells drives resistance to tumor immunotherapy. JCI Insight.

[89] Ringelhan M, Pfister D, O'Connor T, Pikarsky E, Heikenwalder M (2018). The immunology of hepatocellular carcinoma. Nat Immunol.

[90] Wu QC, Zhou WH, Yin SY, Zhou Y, Chen TC, Qian JJ, Su R, Hong LJ, Lu HH, Zhang F (2019). Blocking triggering receptor expressed on myeloid Cells-1-positive tumor-associated macrophages induced by hypoxia reverses immunosuppression and anti-programmed cell death ligand 1 resistance in liver Cancer. Hepatology.

[91] Sun ZR, Du CC, Xu PB, Miao CH (2019). Surgical trauma-induced CCL18 promotes recruitment of regulatory T cells and colon cancer progression. J Cell Physiol.

[92] Jing WQ, Guo X, Wang GY, Bi YX, Han LH, Zhu QF, Qiu CH, Tanaka M, Zhao YX (2020). Breast cancer cells promote CD169(+) macrophage-associated immunosuppression through JAK2-mediated PD-L1 upregulation on macrophages. Int Immunopharmacol.

[93] Fan CS, Chen LL, Hsu TA, Chen CC, Chua KV, Li CP, Huang TS (2019). Endothelial-mesenchymal transition harnesses HSP90 alpha-secreting M2-macrophages to exacerbate pancreatic ductal adenocarcinoma. J Hematol Oncol.

[94] Barkal AA, Brewer RE, Markovic M, Kowarsky M, Barkal SA, Zaro BW, Krishnan V, Hatakeyama J, Dorigo O, Barkal LJ, Weissman IL (2019). CD24 signalling through macrophage Siglec-10 is a target for cancer immunotherapy. Nature.

[95] Barkal AA, Weiskopf K, Kao KS, Gordon SR, Rosental B, Yiu YY, George BM, Markovic M, Ring NG, Tsai JM (2018). Engagement of MHC class I by the inhibitory receptor LILRB1 suppresses macrophages and is a target of cancer immunotherapy. Nat Immunol.

[96] Reis ES, Mastellos DC, Ricklin D, Mantovani A, Lambris JD (2018). Complement in cancer: untangling an intricate relationship. Nat Rev Immunol.

[97] Paulus P, Stanley ER, Schafer R, Abraham D, Aharinejad S (2006). Colony-stimulating factor-1 antibody reverses chemoresistance in human MCF-7 breast cancer xenografts. Cancer Res.

[98] Halbrook CJ, Pontious C, Kovalenko I, Lapienyte L, Dreyer S, Lee HJ, Thurston G, Zhang YQ, Lazarus J, Sajjakulnukit P (2019). Macrophage-released pyrimidines inhibit gemcitabine therapy in pancreatic Cancer. Cell Metab.

[99] Buchholz SM, Goetze RG, Singh SK, Ammer-Herrmenau C, Richards FM, Jodrell DI, Buchholz M, Michl P, Ellenrieder V, Hessmann E, Neesse A (2020). Depletion of macrophages improves therapeutic response to gemcitabine in murine pancreas Cancer. Cancers.

[100] Dong XL, Sun RM, Wang J, Yu SZ, Cui JQ, Guo Z, Pan XH, Sun J, Yang J, Pan LL (2020). Glutathione S-transferases P1-mediated interleukin-6 in tumor-associated macrophages augments drug-resistance in MCF-7 breast cancer. Biochem Pharmacol.

[101] Kuwada K, Kagawa S, Yoshida R, Sakamoto S, Ito A, Watanabe M, Ieda T, Kuroda S, Kikuchi S, Tazawa H, Fujiwara T (2018). The epithelial-to-mesenchymal transition induced by tumor-associated macrophages confers chemoresistance in peritoneally disseminated pancreatic cancer. J Exp Clin Cancer Res.

[102] Dudas J, Ladanyi A, Ingruber J, Steinbichler TB, Riechelmann H (2020). Epithelial to mesenchymal transition: a mechanism that fuels Cancer radio/Chemoresistance. Cells.

[103] Li DB, Ji HF, Niu XJ, Yin L, Wang YR, Gu YC, Wang JL, Zhou XP, Zhang H, Zhang QY (2020). Tumor-associated macrophages secrete CC-chemokine ligand 2 and induce tamoxifen resistance by activating PI3K/Akt/mTOR in breast cancer. Cancer Sci.

[104] Jeong H, Kim S, Hong BJ, Lee CJ, Kim YE, Bok S, Oh JM, Gwak SH, Yoo MY, Lee MS (2019). Tumor-associated macrophages enhance tumor hypoxia and aerobic glycolysis. Cancer Res.

[105] Binenbaum Y, Fridman E, Yaari Z, Milman N, Schroeder A, Ben David G, Shlomi T, Gil Z (2018). Transfer of miRNA in macrophage-derived exosomes induces drug resistance in pancreatic adenocarcinoma. Cancer Res.

[106] Ma YS, Wu TM, Ling CC, Yu F, Zhang J, Cao PS, Gu LP, Wang HM, Xu H, Li L (2021). M2 macrophage-derived exosomal microRNA-155-5p promotes the immune escape of colon cancer by downregulating ZC3H12B. Mol Ther Oncolytics.

[107] Sahraei M, Chaube B, Liu YT, Sun J, Kaplan A, Price NL, Ding W, Oyaghire S, Garcia-Milian R, Mehta S (2019). Suppressing miR-21 activity in tumor-associated macrophages promotes an antitumor immune response. J Clin Investig.

[108] Nowak M, Klink M (2020). The role of tumor-associated macrophages in the progression and Chemoresistance of ovarian Cancer. Cells.

[109] Zhang QW, Liu L, Gong CY, Shi HS, Zeng YH, Wang XZ, Zhao YW, Wei YW (2012). Prognostic significance of tumor-associated macrophages in solid tumor: a Meta-analysis of the literature. PLoS One.

[110] Ding W, Tan YL, Qian Y, Xue WB, Wang YB, Jiang P, Xu XZ (2019). Clinicopathologic and prognostic significance of tumor-associated macrophages in patients with hepatocellular carcinoma: a meta-analysis. PLoS One.

[111] Mei JD, Xiao ZL, Guo CL, Pu Q, Ma L, Liu CW, Lin F, Liao H, You ZB, Liu LX (2016). Prognostic impact of tumor-associated macrophage infiltration in non-small cell lung cancer: a systemic review and meta-analysis. Oncotarget.

[112] Kumar AT, Knops A, Swendseid B, Martinez-Outschoom U, Harshyne L, Philp N, Rodeck U, Luginbuhl A, Cognetti D, Johnson J, Curry J (2019). Prognostic significance of tumor-associated macrophage content in head and neck squamous cell carcinoma: a Meta-analysis. Front Oncol.

[113] Kubler K, Ayub TH, Weber SK, Zivanovic O, Abramian A, Keyver-Paik MD, Mallmann MR, Kaiser C, Serce NB, Kuhn W, Rudlowski C (2014). Prognostic significance of tumor-associated macrophages in endometrial adenocarcinoma. Gynecol Oncol.

[114] Larson RC, Maus MV (2021). Recent advances and discoveries in the mechanisms and functions of CAR T cells. Nat Rev Cancer.

[115] Romero D (2016). CAR T cells ready to go mainstream. *Nature reviews*. Clin Oncol.

[116] Martinez M, Moon EK (2019). CAR T cells for solid tumors: new strategies for finding, infiltrating, and surviving in the tumor microenvironment. Front Immunol.

[117] Roghanian A, Stopforth RJ, Dahal LN, Cragg MS (2018). New revelations from an old receptor: immunoregulatory functions of the inhibitory fc gamma receptor, FcRIIB (CD32B). J Leukoc Biol.

[118] Morrissey MA, Williamson AP, Steinbach AM, Roberts EW, Kern N, Headley MB, Vale RD (2018). Chimeric antigen receptors that trigger phagocytosis. Elife.

[119] Zhang WL, Liu L, Su HF, Liu Q, Shen J, Dai HR, Zheng W, Lu Y, Zhang WJ, Bei YC, Shen PP (2019). Chimeric antigen receptor macrophage therapy for breast tumours mediated by targeting the tumour extracellular matrix. Br J Cancer.

[120] Klichinsky M, Ruella M, Shestova O, Lu XM, Best A, Zeeman M, Schmierer M, Gabrusiewicz K, Anderson NR, Petty NE (2020). Human chimeric antigen receptor macrophages for cancer immunotherapy. Nat Biotechnol.

[121] Zhang L, Tian L, Dai XY, Yu H, Wang JJ, Lei AH, Zhu MM, Xu JP, Zhao W, Zhu YQ (2020). Pluripotent stem cell-derived CAR-macrophage cells with antigen-dependent anti-cancer cell functions. J Hematol Oncol.

[122] Niu ZY, Chen GX, Chang W, Sun PY, Luo ZX, Zhang HY, Zhi LT, Guo CJ, Chen H, Yin MC, Zhu WL (2021). Chimeric antigen receptor-modified macrophages trigger systemic anti-tumour immunity. J Pathol.

[123] Aalipour A, Chuang HY, Murty S, D'Souza AL, Park SM, Gulati GS, Patel CB, Beinat C, Simonetta F, Martinic I (2019). Engineered immune cells as highly sensitive cancer diagnostics. Nat Biotechnol.

[124] Villanueva MT (2020). Macrophages get a CAR. Nat Rev Immunol.

[125] Ribas A, Wolchok JD (2018). Cancer immunotherapy using checkpoint blockade. Science.

[126] Lebbe C, Weber JS, Maio M, Neyns B, Harmankaya K, Hamid O, O'Day SJ, Konto C, Cykowski L, McHenry MB, Wolchok JD (2014). Survival follow-up and ipilimumab retreatment of patients with advanced melanoma who received ipilimumab in prior phase II studies. Ann Oncol.

[127] Shen S, Zhang Y, Chen KG, Luo YL, Wang J (2018). Cationic polymeric nanoparticle delivering CCR2 siRNA to inflammatory monocytes for tumor microenvironment modification and Cancer therapy. Mol Pharm.

[128] Trac N, Chen LY, Zhang AL, Liao CP, Poon C, Wang J, Ando Y, Joo J, Garri C, Shen KY (2021). CCR2-targeted micelles for anti-cancer peptide delivery and immune stimulation. J Control Release.

[129] Zhang XH, Detering L, Sultan D, Luehmann H, Li L, Heo GS, Zhang XL, Lou LL, Grierson PM, Greco S (2021). CC chemokine receptor 2-targeting copper nanoparticles for positron emission tomography-guided delivery of gemcitabine for pancreatic ductal adenocarcinoma. ACS Nano.

[130] Jung K, Heishi T, Khan OF, Kowalski PS, Incio J, Rahbari NN, Chung E, Clark JW, Willett CG, Luster AD (2017). Ly6Clo monocytes drive immunosuppression and confer resistance to anti-VEGFR2 cancer therapy. J Clin Invest.

[131] Shen S, Li HJ, Chen KG, Wang YC, Yang XZ, Lian ZX, Du JZ, Wang J (2017). Spatial targeting of tumor-associated macrophages and tumor cells with a pH-sensitive cluster Nanocarrier for Cancer Chemoimmunotherapy. Nano Lett.

[132] Qian Y, Qiao S, Dai YF, Xu GQ, Dai BL, Lu LS, Yu X, Luo QM, Zhang ZH (2017). Molecular-targeted immunotherapeutic strategy for melanoma via dual-targeting nanoparticles delivering small interfering RNA to tumor-associated macrophages. ACS Nano.

[133] Wang YC, Luan ZY, Zhao CY, Bai CH, Yang KJ (2020). Target delivery selective CSF-1R inhibitor to tumor-associated macrophages via erythrocyte-cancer cell hybrid membrane camouflaged pH-responsive copolymer micelle for cancer immunotherapy. Eur J Pharm Sci.

[134] Wei Q, Shen N, Yu HY, Wang Y, Tang ZH, Chen XS (2020). FXIIIa substrate peptide decorated BLZ945 nanoparticles for specifically remodeling tumor immunity. Biomater Sci.

[135] Tian LL, Yi X, Dong ZL, Xu J, Liang C, Chao Y, Wang YX, Yang K, Liu Z (2018). Calcium bisphosphonate nanoparticles with Chelator-free radiolabeling to deplete tumor-associated macrophages for enhanced Cancer radioisotope therapy. ACS Nano.

[136] Zang XL, Zhang XX, Hu HY, Qiao MX, Zhao XL, Deng YH, Chen DW (2019). Targeted delivery of Zoledronate to tumor-associated macrophages for Cancer immunotherapy. Mol Pharm.

[137] Zhang XX, Zang XL, Qiao MX, Zhao XL, Hu HY, Chen DW (2020). Targeted delivery of Dasatinib to deplete tumor-associated macrophages by Mannosylated mixed micelles for tumor immunotherapy. Acs Biomater Sci Eng.

[138] Liu Y, Wang J, Zhang J, Marbach S, Xu W, Zhu L (2020). Targeting tumor-associated macrophages by MMP2-sensitive apoptotic body-mimicking nanoparticles. ACS Appl Mater Interfaces.

[139] Deng CF, Zhang Q, Jia MD, Zhao J, Sun X, Gong T, Zhang ZR (2019). Tumors and their microenvironment dual-targeting chemotherapy with local immune adjuvant therapy for effective antitumor immunity against breast Cancer. Adv Sci.

[140] Tian DD, Qin FF, Zhao HJ, Zhang CF, Wang H, Liu N, Ai YQ (2021). Bio-responsive nanoparticle for tumor targeting and enhanced photo-immunotherapy. Colloids Surf B Biointerfaces.

[141] Huang ZS, Yao D, Ye QS, Jiang HJ, Gu R, Ji CW, Wu JH, Hu YQ, Yuan A (2021). Zoledronic acid-gadolinium coordination polymer Nanorods for improved tumor Radioimmunotherapy by Synergetically inducing immunogenic cell death and reprogramming the immunosuppressive microenvironment. ACS Nano.

[142] Yu GT, Rao L, Wu H, Yang LL, Bu LL, Deng WW, et al. Myeloid-derived suppressor cell membrane-coated magnetic nanoparticles for Cancer Theranostics by inducing macrophage polarization and synergizing immunogenic cell death. Adv Funct Mater. 2018;28:1801389.

[143] Rong L, Zhang Y, Li WS, Su ZG, Fadhil JI, Zhang C (2019). Iron chelated melanin-like nanoparticles for tumor-associated macrophage repolarization and cancer therapy. Biomaterials.

[144] Rodell CB, Arlauckas SP, Cuccarese MF, Garris CS, Ahmed RLMS, Kohler RH, Pittet MJ, Weissleder R (2018). TLR7/8-agonist-loaded nanoparticles promote the polarization of tumour-associated macrophages to enhance cancer immunotherapy. Nat Biomed Eng.

[145] Rodell CB, Ahmed MS, Garris CS, Pittet MJ, Weissleder R (2019). Development of Adamantane-conjugated TLR7/8 agonists for supramolecular delivery and Cancer immunotherapy. Theranostics.

[146] Shan H, Dou WL, Zhang Y, Qi M (2020). Targeted ferritin nanoparticle encapsulating CpG oligodeoxynucleotides induces tumor-associated macrophage M2 phenotype polarization into M1 phenotype and inhibits tumor growth. Nanoscale.

[147] Bolli E, Scherger M, Arnouk SM, Antunes ARP, Strassburger D, Urschbach M, Stickdorn J, De Vlaminck K, Movahedi K, Rader HJ (2021). Targeted repolarization of tumor-associated macrophages via Imidazoquinoline-linked Nanobodies. Adv Sci.

[148] Liu LQ, Wang Y, Guo X, Zhao JY, Zhou SB (2020). A biomimetic polymer magnetic Nanocarrier polarizing tumor-associated macrophages for potentiating immunotherapy. Small.

[149] Li H, Somiya M, Kuroda S (2021). Enhancing antibody-dependent cellular phagocytosis by re-education of tumor-associated macrophages with resiquimod-encapsulated liposomes. Biomaterials.

[150] Nie WD, Wu GH, Zhang JF, Huang LL, Ding JJ, Jiang AQ, Zhang YH, Liu YH, Li JC, Pu KY, Xie HY (2020). Responsive exosome Nano-bioconjugates for synergistic Cancer therapy. Angew Chem Int Ed Engl.

[151] Rao L, Zhao SK, Wen CR, Tian R, Lin LS, Cai B, Sun Y, Kang F, Yang Z, He LC (2020). Activating macrophage-mediated Cancer immunotherapy by genetically edited nanoparticles. Adv Mater.

[152] Chen Q, Wang C, Zhang XD, Chen GJ, Hu QY, Li HJ, Wang JQ, Wen D, Zhang YQ, Lu YF (2019). In situ sprayed bioresponsive immunotherapeutic gel for post-surgical cancer treatment. Nat Nanotechnol.

[153] Li CX, Zhang Y, Dong X, Zhang L, Liu MD, Li B, Zhang MK, Feng J, Zhang XZ (2019). Artificially reprogrammed macrophages as tumor-tropic immunosuppression-resistant biologics to realize therapeutics production and immune activation. Adv Mater.

[154] Li K, Lu L, Xue CC, Liu J, He Y, Zhou J, Xia ZZL, Dai LL, Luo Z, Mao YL, Cai KY (2020). Polarization of tumor-associated macrophage phenotype via porous hollow iron nanoparticles for tumor immunotherapy in vivo. Nanoscale.

[155] Gao F, Tang Y, Liu WL, Zou MZ, Huang C, Liu CJ, Zhang XZ (2019). Intra/extracellular lactic acid exhaustion for synergistic metabolic therapy and immunotherapy of tumors. Adv Mater.

[156] Chang CC, Dinh TK, Lee YA, Wang FN, Sung YC, Yu PL, Chiu SC, Shih YC, Wu CY, Huang YD (2020). Nanoparticle delivery of MnO2 and antiangiogenic therapy to overcome hypoxia-driven tumor escape and suppress hepatocellular carcinoma. ACS Appl Mater Interfaces.

[157] Xu JJ, Zheng BB, Zhang SH, Liao XL, Tong QL, Wei GG, et al. Copper sulfide nanoparticle-redirected macrophages for adoptive transfer therapy of melanoma. Adv Funct Mater. 2021;31:2008022.

[158] Hou T, Wang TQ, Mu WW, Yang R, Liang S, Zhang ZP, Fu SL, Gao T, Liu YJ, Zhang N (2021). Nanoparticle-loaded polarized-macrophages for enhanced tumor targeting and cell-chemotherapy. Nanomicro Lett.

[159] Liu T, Xu LG, He LZ, Zhao JF, Zhang ZH, Chen Q, et al. Selenium nanoparticles regulates selenoprotein to boost cytokine-induced killer cells-based cancer immunotherapy. Nano Today. 2020;35:100975.

[160] Shobaki N, Sato Y, Suzuki Y, Okabe N, Harashima H (2020). Manipulating the function of tumor-associated macrophages by siRNA-loaded lipid nanoparticles for cancer immunotherapy. J Control Release.

[161] Esser AK, Ross MH, Fontana F, Su XM, Gabay A, Fox GC, Xu YL, Xiang JY, Schmieder AH, Yang XX (2020). Nanotherapy delivery of c-myc inhibitor targets Protumor macrophages and preserves antitumor macrophages in breast Cancer. Theranostics.

[162] Ramesh A, Kumar S, Nandi D, Kulkarni A (2019). CSF1R-and SHP2-inhibitor-loaded nanoparticles enhance cytotoxic activity and phagocytosis in tumor-associated macrophages. Adv Mater.

[163] Ramesh A, Brouillard A, Kumar S, Nandi D, Kulkarni A (2020). Dual inhibition of CSF1R and MAPK pathways using supramolecular nanoparticles enhances macrophage immunotherapy. Biomaterials.

[164] Wang Y, Tiruthani K, Li SR, Hu MY, Zhong GJ, Tang Y, Roy S, Zhang LL, Tan J, Liao CH, Liu RH (2021). mRNA delivery of a bispecific single-domain antibody to polarize tumor-associated macrophages and synergize immunotherapy against liver malignancies. Adv Mater.

[165] He YH, Wang MN, Li XL, Yu T, Gao X (2020). Targeted MIP-3 beta plasmid nanoparticles induce dendritic cell maturation and inhibit M2 macrophage polarisation to suppress cancer growth. Biomaterials.

[166] Zhang YM, Guo C, Liu LP, Xu J, Jiang H, Li DQ, Lan JJ, Li J, Yang J, Tu QM (2020). ZnO-based multifunctional nanocomposites to inhibit progression and metastasis of melanoma by eliciting antitumor immunity via immunogenic cell death. Theranostics.

[167] Yang SN, Zhang YM, Lu SJ, Yang L, Yu SN, Yang HY (2021). CaCO3-encapsulated au nanoparticles modulate macrophages toward M1-like phenotype. Acs Applied Bio Mater.

[168] Rangasami VK, Samanta S, Parihar VS, Asawa K, Zhu K, Varghese OP, Teramura Y, Nilsson B, Hilborn J, Harris RA, Oommen OP (2021). Harnessing hyaluronic acid-based nanoparticles for combination therapy: a novel approach for suppressing systemic inflammation and to promote antitumor macrophage polarization. Carbohydr Polym.

[169] Han S, Wang W, Wang S, Yang T, Zhang G, Wang D, Ju R, Lu Y, Wang H, Wang L (2021). Tumor microenvironment remodeling and tumor therapy based on M2-like tumor associated macrophage-targeting nano-complexes. Theranostics.

[170] Hu MY, Wang Y, Xu LG, An S, Tang Y, Zhou XF, Li JJ, Liu RH, Huang LA (2019). Relaxin gene delivery mitigates liver metastasis and synergizes with check point therapy. Nat Commun.

[171] Fu XC, Yu JM, Yuan AR, Liu LB, Zhao H, Huang YM, Shen S, Lv FT, Wang S (2021). Polymer nanoparticles regulate macrophage repolarization for antitumor treatment. Chem Commun.

[172] Li L, Zhen MM, Wang HY, Sun ZH, Jia W, Zhao ZP, Zhou C, Liu S, Wang CR, Bai CL (2020). Functional Gadofullerene nanoparticles trigger robust Cancer immunotherapy based on rebuilding an immunosuppressive tumor microenvironment. Nano Lett.

[173] Chen YZ, Song WT, Shen LM, Qiu NS, Hu MY, Liu Y, Liu Q, Huang L (2019). Vasodilator hydralazine promotes nanoparticle penetration in advanced desmoplastic tumors. ACS Nano.

[174] Huang W, He LZ, Ouyang J, Chen Q, Liu C, Tao W, Chen TF (2020). Triangle-shaped tellurium Nanostars potentiate radiotherapy by boosting checkpoint blockade immunotherapy. Matter.

[175] Deng GJ, Sun ZH, Li SP, Peng XH, Li WJ, Zhou LH, Ma YF, Gong P, Cai LT (2018). Cell-membrane immunotherapy based on natural killer cell membrane coated nanoparticles for the effective inhibition of primary and Abscopal tumor growth. ACS Nano.

[176] Argyle D, Kitamura T (2018). Targeting macrophage-recruiting chemokines as a novel therapeutic strategy to prevent the progression of solid tumors. Front Immunol.

[177] Pyonteck SM, Akkari L, Schuhmacher AJ, Bowman RL, Sevenich L, Quail DF, Olson OC, Quick ML, Huse JT, Teijeiro V (2013). CSF-1R inhibition alters macrophage polarization and blocks glioma progression. Nat Med.

[178] Halama N, Zoernig I, Berthel A, Kahlert C, Klupp F, Suarez-Carmona M, Suetterlin T, Brand K, Krauss J, Lasitschka F (2016). Tumoral immune cell exploitation in colorectal Cancer metastases can be targeted effectively by anti-CCR5 therapy in Cancer patients. Cancer Cell.

[179] Tap WD, Wainberg ZA, Anthony SP, Ibrahim PN, Zhang C, Healey JH, Chmielowski B, Staddon AP, Cohn AL, Shapiro GI (2015). Structure-guided blockade of CSF1R kinase in Tenosynovial Giant-cell tumor. N Engl J Med.

[180] Yan D, Kowal J, Akkari L, Schuhmacher AJ, Huse JT, West BL, Joyce JA (2017). Inhibition of colony stimulating factor-1 receptor abrogates microenvironment-mediated therapeutic resistance in gliomas. Oncogene.

[181] Xia Q, Zhang YT, Li Z, Hou XF, Feng NP (2019). Red blood cell membrane-camouflaged nanoparticles: a novel drug delivery system for antitumor application. Acta Pharm Sin B.

[182] Stresing V, Daubine F, Benzaid I, Monkkonen H, Clezardin P (2007). Bisphosphonates in cancer therapy. Cancer Lett.

[183] Yu SS, Lau CM, Barham WJ, Onishko HM, Nelson CE, Li HM, Smith CA, Yull FE, Duvall CL, Giorgio TD (2013). Macrophage-specific RNA interference targeting via “click”, Mannosylated polymeric micelles. Mol Pharm.

[184] Zhang L, Zhou HL, Belzile O, Thorpe P, Zhao DW (2014). Phosphatidylserine-targeted bimodal liposomal nanoparticles for in vivo imaging of breast cancer in mice. J Control Release.

[185] Vandenbroucke RE, Libert C (2014). Is there new hope for therapeutic matrix metalloproteinase inhibition?. Nat Rev Drug Discov.

[186] Rodriguez-Ruiz ME, Vitale I, Harrington KJ, Melero I, Galluzzi L (2020). Immunological impact of cell death signaling driven by radiation on the tumor microenvironment. Nat Immunol.

[187] Deutsch E, Chargari C, Galluzzi L, Kroemer G (2019). Optimising efficacy and reducing toxicity of anticancer radioimmunotherapy. Lancet Oncol.

[188] Farzin A, Etesami SA, Quint J, Memic A, Tamayol A (2020). Magnetic nanoparticles in Cancer therapy and diagnosis. Adv Healthc Mater.

[189] Zhang F, Lu GH, Wen XL, Li F, Ji XY, Li QQ, Wu MY, Cheng QZ, Yu YK, Tang J, Mei L (2020). Magnetic nanoparticles coated with polyphenols for spatio-temporally controlled cancer photothermal/immunotherapy. J Control Release.

[190] Shime H, Matsumoto M, Oshiumi H, Tanaka S, Nakane A, Iwakura Y, Tahara H, Inoue N, Seya T (2012). Toll-like receptor 3 signaling converts tumor-supporting myeloid cells to tumoricidal effectors. Proc Natl Acad Sci U S A.

[191] Joshi S, Singh AR, Zulcic M, Durden DL (2014). A macrophage-dominant PI3K isoform controls hypoxia-induced HIF1 alpha and HIF2 alpha stability and tumor growth, angiogenesis, and metastasis. Mol Cancer Res.

[192] Yang GB, Xu LG, Chao Y, Xu J, Sun XQ, Wu YF, Peng R, Liu Z (2017). Hollow MnO2 as a tumor-microenvironment-responsive biodegradable nano-platform for combination therapy favoring antitumor immune responses. Nat Commun.

[193] Stepanov AV, Markov OV, Chernikov IV, Gladkikh DV, Zhang HK, Jones T, Sen'kova AV, Chernolovskaya EL, Zenkova MA, Kalinin RS (2018). Autocrine-based selection of ligands for personalized CAR-T therapy of lymphoma. Sci Adv.

[194] Wei BC, Pan JM, Yuan RT, Shao BF, Wang Y, Guo X, Zhou SB (2021). Polarization of tumor-associated macrophages by nanoparticle-loaded Escherichia coli combined with immunogenic cell death for Cancer immunotherapy. Nano Lett.

[195] Yu H, Kortylewski M, Pardoll D (2007). Crosstalk between cancer and immune cells: role of STAT3 in the tumour microenvironment. Nat Rev Immunol.

[196] Liu LL, Lu Y, Martinez J, Bi YJ, Lian GJ, Wang TT, Milasta S, Wang J, Yang M, Liu GW (2016). Proinflammatory signal suppresses proliferation and shifts macrophage metabolism from Myc-dependent to HIF1 alpha-dependent. Proc Natl Acad Sci U S A.

[197] Cannarile MA, Weisser M, Jacob W, Jegg AM, Ries CH, Ruttinger D (2017). Colony-stimulating factor 1 receptor (CSF1R) inhibitors in cancer therapy. J Immunother Cancer.

[198] Zhou DX, Huang C, Lin Z, Zhan SX, Kong LN, Fang CB, Li J (2014). Macrophage polarization and function with emphasis on the evolving roles of coordinated regulation of cellular signaling pathways. Cell Signal.

[199] Svensson S, Abrahamsson A, Rodriguez GV, Olsson AK, Jensen L, Cao YH, Dabrosin C (2015). CCL2 and CCL5 are novel therapeutic targets for estrogen-dependent breast Cancer. Clin Cancer Res.

[200] Bergamaschi A, Tagliabue E, Sorlie T, Naurne B, Triulzi T, Orlandi R, Russnes HG, Nesland JM, Tammi R, Auvinen P (2008). Extracellular matrix signature identifies breast cancer subgroups with different clinical outcome. J Pathol.

[201] Georgoudaki AM, Prokopec KE, Boura VF, Hellqvist E, Sohn S, Ostling J, Dahan R, Harris RA, Rantalainen M, Klevebring D (2016). Reprogramming tumor-associated macrophages by antibody targeting inhibits Cancer progression and metastasis. Cell Rep.

[202] Yue YL, Li FF, Li Y, Wang YZ, Guo XJ, Cheng ZX, Li N, Ma XT, Nie GJ, Zhao X (2021). Biomimetic nanoparticles carrying a repolarization agent of tumor-associated macrophages for remodeling of the inflammatory microenvironment following Photothermal therapy. ACS Nano.

[203] Chen CL, Song MY, Du YY, Yu Y, Li CG, Han Y, Yan F, Shi Z, Feng SH (2021). Tumor-associated-macrophage-membrane-coated nanoparticles for improved photodynamic immunotherapy. Nano Lett.

[204] Rodell CB, Koch PD, Weissleder R (2019). Screening for new macrophage therapeutics. Theranostics.

